# Assessment of Essential and Toxic Element Levels in Endometrial and Ovarian Cancer

**DOI:** 10.3390/cancers18071051

**Published:** 2026-03-24

**Authors:** Paweł Ordon, Kacper Boroń, Krzysztof Bereza, Dariusz Boroń, Piotr Ossowski, Tomasz Sirek, Agata Sirek, Wojciech Kulej, Grzegorz Wyrobiec, Beniamin Oskar Grabarek

**Affiliations:** 1 Collegium Medicum, WSB University, 41-300 Dabrowa Gornicza, Poland; dariusz@boron.pl (D.B.); drpiotrossowski15@gmail.com (P.O.); drtskierka@gmail.com (T.S.); sirek.agata@gmail.com (A.S.); wojciechkulej87@gmail.com (W.K.); bgrabarek7@gmail.com (B.O.G.); 2 Department of Plastic Surgery, Faculty of Medicine, Academia of Silesia, 40-555 Katowice, Poland; q375@icloud.com; 3 Department of Mother and Child Health, Faculty of Health Sciences, Institute of Nursing and Midwifery, Jagiellonian University Medical College, 31-008 Cracow, Poland; kbereza06@gmail.com; 4 Faculty of Medicine and Health Sciences, Andrzej Frycz Modrzewski University in Kraków, 30-705 Cracow, Poland; 5 Department of Gynecology and Obstetrics, TOMMED Specjalisci od Zdrowia, 40-851 Katowice, Poland; 6 Department of Gynecology and Obstetrics with Gynecologic Oncology, Ludwik Rydygier Memorial Specialized Hospital, 31-826 Kraków, Poland; 7 Department of Plastic and Reconstructive Surgery, Hospital for Minimally Invasive and Reconstructive Surgery in Bielsko-Biała, 43-316 Bielsko-Biala, Poland; 8 Department of Histology and Cell Pathology in Zabrze, Faculty of Medical Sciences in Zabrze, Medical University of Silesia in Katowice, 41-808 Zabrze, Poland; gwyrobiec@sum.edu.pl

**Keywords:** trace elements, ovarian cancer, endometrioid endometrial cancer, inductively coupled plasma–optical emission spectrometry (ICP-OES)

## Abstract

Background/Objectives: Endometrial cancer (EC) is a multifactorial disease influenced by metabolic, hormonal, and environmental factors. Trace and macroelements play a critical role in cellular homeostasis, oxidative stress, and tumor progression; however, their relationship with EC grading and clinical characteristics remains insufficiently understood. Methods: This study evaluated the concentrations of selected macro- and trace elements (Na, K, Ca, P, Mg, Mn, Cu, Zn, Be, As, Cr, Mo, Ti, Tl, Pb) in patients with endometrial cancer (G1–G3) and a control group (C). Elemental analysis was performed using inductively coupled plasma optical emission spectrometry (ICP-OES). Associations between elemental concentrations and clinicopathological variables, including age, body mass index (BMI), menopausal status, diabetes, and smoking, were assessed using appropriate statistical tests, including ANOVA with Tukey’s post hoc analysis and Student’s *t*-test. Multivariate regression analysis was performed to identify independent predictors of elemental alterations. Results: Significant differences in elemental concentrations were observed across EC grading. Higher-grade tumors were associated with increased levels of Ca, P, Mg, and Mn, while Na and K showed a decreasing trend with tumor progression. No statistically significant differences were observed for Zn, Ti, Tl, or Pb across histological grades. Stratified analyses demonstrated that clinical and metabolic factors had a limited and selective impact on elemental profiles. Age and BMI were associated with minor variations in selected elements, whereas menopausal status, diabetes, and smoking showed predominantly non-significant or inconsistent effects. Multivariate analysis identified histological grade as the primary determinant of elemental alterations, while other variables exhibited weaker or element-specific associations. Conclusions: Elemental homeostasis in endometrial cancer is primarily associated with tumor progression rather than systemic metabolic or lifestyle factors. Changes in Ca-, P-, Mg-, and Mn-related pathways may reflect tumor-driven metabolic reprogramming, whereas most trace elements remain relatively stable. These findings suggest that elemental profiling may provide insight into EC biology, although its clinical utility requires further investigation.

## Simple Summary

Endometrial and ovarian cancers are among the most common and serious gynecological malignancies affecting women worldwide. Increasing evidence suggests that imbalances in essential and toxic elements within the body may influence cancer development and progression. In this study, we analyzed the concentrations of selected essential elements (such as sodium, magnesium, copper, and zinc) and toxic elements (including lead and thallium) in cancerous and non-cancerous tissues from patients with endometrial and ovarian cancer. Using advanced elemental analysis techniques, we evaluated the relationships between tissue metal levels and tumor severity and clinical–metabolic factors. Our findings show that more advanced tumors tend to accumulate certain toxic metals while losing important essential elements involved in normal cellular metabolism. These results suggest that disturbances in metal balance are associated with tumor biology and may reflect disease-related metabolic alterations.

## 1. Introduction

Endometrial cancer (EC) remains the most frequently diagnosed gynecological malignancy in developed countries, and its incidence continues to increase steadily [1,2]. Most cases are detected at an early stage due to the presence of abnormal uterine bleeding—particularly postmenopausal bleeding—although this symptom lacks specificity and is observed in a wide range of benign endometrial conditions [3,4]. A well-established constellation of risk factors for EC includes obesity, diabetes, metabolic syndrome, prolonged exposure to unopposed estrogens, early menarche, late menopause, infertility, and chronic anovulation [5,6]. Genetic predispositions such as Lynch syndrome and Cowden syndrome further elevate risk [7,8]. While the historical Bokhman classification divided EC into estrogen-dependent (type I) and estrogen-independent (type II) tumors, modern molecular taxonomies, including the TCGA system, have shifted clinical practice toward biologically defined subgroups [9,10,11]. Despite these advances, reliable biomarkers for early diagnosis and risk stratification remain lacking.

Ovarian cancer (OC), although less common, is the most lethal gynecological cancer due to its asymptomatic onset, extensive intra-abdominal spread at diagnosis, and limited screening strategies [12,13,14]. Its multifactorial pathogenesis encompasses genetic, hormonal, environmental, and metabolic influences [15,16]. Understanding modifiable environmental contributors to both EC and OC is therefore of major clinical relevance.

Among environmental factors implicated in carcinogenesis, exposure to essential and toxic elements has received growing attention [17,18,19,20]. Essential elements such as sodium (Na), potassium (K), calcium (Ca), phosphorus (P), magnesium (Mg), manganese (Mn), copper (Cu), and zinc (Zn) play crucial roles in enzymatic activity, redox balance, DNA stability, and cellular signaling. Their concentrations in tissues are tightly regulated, and even subtle disturbances may influence oxidative stress, cell proliferation, and inflammatory responses [21,22,23]. Conversely, toxic elements—including lead (Pb), arsenic (As), chromium (Cr), beryllium (Be), thallium (Tl), titanium (Ti), and molybdenum (Mo)—may disrupt cellular homeostasis and contribute to carcinogenic processes [24,25,26,27]. Several of these elements have been classified as carcinogenic or potentially carcinogenic to humans by the International Agency for Research on Cancer (IARC) [28].

Toxic elements may exert their effects through multiple mechanisms, including induction of oxidative stress, interference with DNA repair systems, disruption of apoptosis, and modulation of signaling pathways [29,30]. In addition, certain metals can act as metalloestrogens, binding to and activating estrogen receptors, thereby mimicking estrogenic signaling—a mechanism particularly relevant in hormonally driven malignancies such as EC and OC [31,32].

Environmental exposure to such elements varies depending on geography, diet, and industrial or occupational factors. Importantly, tissue-based measurements provide a more accurate reflection of local metal accumulation compared to circulating biomarkers, which often represent only recent exposure [33,34]. Cancer tissues may accumulate or redistribute elements differently than adjacent non-cancerous tissues due to altered metabolism, increased proliferation, angiogenesis, and hypoxic conditions [35]. Therefore, direct elemental profiling of tumor tissue represents a valuable approach to understanding the role of metal dyshomeostasis in carcinogenesis. Disturbances in essential element homeostasis may also contribute to tumor development and progression. Elements such as Zn and Cu are involved in antioxidant defense mechanisms, including the activity of Cu/Zn superoxide dismutase [36]. However, imbalances in these and other elements may enhance reactive oxygen species (ROS) generation and promote oxidative damage to cellular structures [37]. Furthermore, interactions between toxic and essential elements may disrupt physiological processes, including enzyme function and DNA repair mechanisms, thereby facilitating malignant transformation [38].

Despite increasing interest in the role of elemental imbalance in cancer biology, data regarding gynecological malignancies remain limited and sometimes inconsistent. Previous studies have suggested that alterations in selected essential and toxic elements may be associated with the risk and progression of EC and OC; however, comprehensive comparative analyses of tumor and non-tumor tissues are still lacking [28,36,39].

Therefore, the aim of this study was to assess the tissue concentrations of selected essential elements (Na, K, Ca, P, Mg, Mn, Cu, Zn) and selected trace/toxic elements (Be, As, Cr, Mo, Ti, Tl, Pb) in malignant and non-malignant endometrial and ovarian tissues using ICP-OES. Particular emphasis was placed on identifying patterns of elemental imbalance associated with tumor progression, including histological grading in endometrial cancer and clinically defined treatment groups in ovarian cancer. In addition, we evaluated the relationships between elemental profiles and key clinical and metabolic factors, such as age, body mass index (BMI), menopausal status, and type 2 diabetes. Given the observational and tissue-based nature of this study, the objective was not to provide an exhaustive characterization of all measured analytes, but rather to identify biologically and clinically relevant trends in metal dyshomeostasis associated with gynecological malignancies.

## 2. Materials and Methods

### 2.1. Material and Methods

The study adhered to the ethical principles outlined in the 2013 Declaration of Helsinki. All procedures involving human participants were conducted with strict attention to data protection and anonymity. Prior to analysis, any information that could allow identification of individual patients was removed from the dataset, ensuring that no participant can be recognized either in the article or in the analytical files. Written informed consent was obtained from every participant. The research protocol received approval from the Bioethical Committee of the Regional Medical Chamber in Kraków (approvals No. 17/KBL/OIL/2024, dated 27 May 2025).

### 2.2. Subjects

#### 2.2.1. Endometrial Cancer Cohort

A total of 70 women undergoing hysterectomy were enrolled in the study. The study group consisted of 50 patients with histopathologically confirmed endometrioid endometrial adenocarcinoma, while the control group included 20 women undergoing surgery for non-oncological indications, such as uterine fibroids or abnormal uterine bleeding unrelated to malignant or premalignant conditions.

All control samples underwent independent histopathological evaluation, confirming the absence of neoplastic, hyperplastic, and inflammatory changes. Only specimens classified as histologically normal endometrium were included in the control group.

To ensure comparability, tissue samples from both cancer and control groups were obtained from anatomically corresponding regions of the uterine corpus. In the cancer group, samples were collected from tumor tissue confirmed by histopathological assessment, whereas in the control group, samples were obtained from morphologically normal endometrium of the uterine body. All specimens were collected intraoperatively during standard surgical procedures and processed under identical conditions prior to elemental analysis.

All participants were older than 45 years. In the study group, the diagnosis of endometrial cancer was established prior to enrollment based on histopathological evaluation of specimens obtained during diagnostic curettage following hysteroscopy. All patients subsequently underwent standard surgical management, including total hysterectomy with pelvic and para-aortic lymphadenectomy.

Exclusion criteria for both study and control groups included endometriosis, adenomyosis, non-endometrioid histological subtypes, adenocarcinoma with squamous differentiation, coexisting cervical carcinoma, hormone therapy within 24 months prior to surgery, morbid obesity (BMI > 40), and a current or past history of other malignancies. Additionally, cases with inflammatory or proliferative endometrial conditions were excluded from the control group. The distribution of these variables is presented in Table 1. Histopathological evaluation enabled tumor classification according to grade: 28 cases were categorized as G1 (well differentiated), 13 as G2 (moderately differentiated), and 9 as G3 (poorly differentiated).

#### 2.2.2. Ovarian Cancer Cohort

The ovarian cancer cohort included 41 women with histologically confirmed ovarian cancer. The majority of cases represented FIGO stages I–III. Only one patient with stage IV disease was identified and was excluded from subgroup analyses to maintain group homogeneity and statistical comparability.

Patients were stratified into two subgroups integrating histopathological tumor type and treatment approach. Group A consisted predominantly of patients with high-grade serous ovarian carcinoma (type II tumors) who underwent surgical treatment followed by standard first-line adjuvant chemotherapy, reflecting more aggressive tumor biology. Group B included patients with mucinous ovarian carcinoma (type I tumors) treated with surgery alone, corresponding to early-stage disease with less aggressive clinical behavior.

Group A (*n* = 28) consisted of patients who underwent surgical treatment followed by standard first-line adjuvant chemotherapy and predominantly represented more advanced-stage and higher-grade tumors (FIGO stages I–III: *n* = 8, 7, and 13, respectively). Group B (*n* = 13) included patients treated with surgery alone and primarily represented early-stage disease (predominantly FIGO stage I) with less aggressive clinicopathological features.

Thus, the applied grouping system reflects clinically relevant categories incorporating tumor histology, disease stage, and treatment strategy, and should be interpreted cautiously as a composite clinical grouping rather than a strictly biological classification.

Histopathological evaluation included assessment of tumor stage, grade, and histological subtype in all cases. Although the cohort exhibited some histological heterogeneity, the majority of tumors were epithelial ovarian carcinomas, with serous and mucinous subtypes predominating in Groups A and B, respectively.

The control group (Group C) comprised 25 women undergoing hysterectomy with bilateral adnexectomy for non-oncological indications. All control ovarian samples were subjected to histopathological verification, confirming normal ovarian architecture without evidence of neoplastic, inflammatory, or proliferative lesions.

To ensure comparability, tissue samples from all groups were collected intraoperatively from anatomically corresponding regions of the ovary and processed under identical conditions prior to elemental analysis. Cases with inflammatory conditions or benign proliferative lesions were excluded from both study and control groups to minimize potential confounding effects on elemental composition. The distribution of these variables is presented in Table 2.

#### 2.2.3. Assessment of Menopause Status, Lifestyle Factors, and Metabolic Conditions

Menopause status, cigarette smoking, BMI, and the presence of type 2 diabetes were recorded for all participants during preoperative evaluation.

Menopause was defined clinically as the absence of menstruation for at least 12 consecutive months not attributable to secondary causes. Smoking status was assessed using a standardized questionnaire and categorized as current smoker or non-smoker.

BMI was calculated as weight in kilograms divided by height in meters squared (kg/m^2^), based on measurements obtained on the day of hospital admission. Participants were categorized as normal weight, overweight, or obese (BMI < 40, in accordance with study exclusion criteria).

Type 2 diabetes was identified based on documented medical history, current use of antidiabetic medication, or laboratory findings consistent with diagnostic criteria, including glycated hemoglobin (HbA1c) ≥ 6.5% and/or fasting plasma glucose ≥ 126 mg/dL measured during preoperative evaluation

#### 2.2.4. Sample Preparation and Quantification of Essential and Toxic Elements

Tissue samples were obtained intraoperatively and immediately processed. Following excision, specimens were thoroughly rinsed with sterile distilled water to remove residual blood, then dried, weighed, and subjected to elemental analysis. The analyzed material consisted of fresh tissue samples that were subsequently dried prior to mineralization; no paraffin-embedded material was used at any stage of sample preparation or analysis.

Approximately 300 mg of each sample was placed in acid-cleaned crucibles and dried at 105 °C for 4–5 h. The dried material was then homogenized and subjected to dry mineralization. For digestion, samples were treated with an ethanol-sulfuric acid mixture (95:5, *v*/*v*) and heated in a muffle furnace with gradual temperature increase up to 550 °C. The obtained ash was dissolved in 3 N hydrochloric acid and diluted to a final volume of 50 mL with deionized water.

Elemental quantification was performed using inductively coupled plasma optical emission spectrometry (ICP-OES) with an Optima 7300 Dual View instrument (PerkinElmer, Waltham, MA, USA). Based on the scope of the present study and the analytes consistently reported throughout the manuscript, the following elements were included in the final analytical panel: Na, K, Ca, P, Mg, Mn, Cu, Zn, Be, As, Cr, Mo, Ti, Tl, and Pb. Earlier broader screening was narrowed to these analytes because they were the elements that met analytical quality criteria and were consistently available for statistical analysis across all study groups.

Calibration curves were prepared using multi-element standard solutions (Merck, Darmstadt, Germany) at a minimum of five concentration levels selected to cover the expected concentration ranges in biological tissues. Calibration linearity was accepted at correlation coefficients (R^2^) of at least 0.999 for all quantified analytes. To minimize matrix effects, calibration standards were prepared in the same acid matrix as the samples. Instrument stability was verified using quality-control standards analyzed after every 10 samples.

Blank correction was performed using reagent blanks processed identically to tissue samples, including all digestion and dilution steps. Blank values were subtracted from the analytical signals prior to final concentration calculations. Limits of detection (LOD) and limits of quantification (LOQ) were determined from repeated blank measurements, with LOD defined as three times the standard deviation of the blank and LOQ as ten times the standard deviation of the blank signal.

Analytical accuracy was assessed using certified reference material for biological tissues (NIST SRM 1577c, Bovine Liver; National Institute of Standards and Technology, Gaithersburg, MD, USA), processed under the same preparation and measurement conditions as the study samples. Although not tissue-identical, this reference material is widely used for validation of trace element determination in biological matrices. Recoveries for the quantified analytes remained within acceptable analytical ranges.

Precision was assessed as both intra-assay and inter-assay variability. Intra-assay precision was evaluated by triplicate analysis of each sample and expressed as relative standard deviation (RSD), which remained below 5%. Inter-assay precision was determined by repeated analysis of selected samples on different days, with RSD values below 8%.

Strict contamination-control procedures were applied throughout the analytical workflow. All glassware and sample vessels were pre-cleaned in 10% nitric acid and thoroughly rinsed with ultrapure deionized water (18.2 MΩ·cm). Sample handling was performed using powder-free gloves and non-metallic instruments whenever possible to minimize exogenous contamination.

ICP-OES measurements were conducted under optimized instrumental conditions, including RF power of approximately 1300–1450 W, plasma gas flow of 15 L/min, auxiliary gas flow of 0.2–0.5 L/min, and nebulizer gas flow of 0.8 L/min. The instrument was operated in dual-view mode (axial and radial), depending on the concentration range of the analyte.

The following analytical emission lines (nm) were used for quantification: Na, 589.592; K, 766.490; Ca, 315.887; P, 213.617; Mg, 285.213; Mn, 257.610; Cu, 324.755; Zn, 213.857; Be, 313.042; As, 188.979; Cr, 267.716; Mo, 203.845; Ti, 334.942; Tl, 190.805; and Pb, 220.353. Final concentrations were recalculated to tissue content based on initial sample mass and dilution volume.

### 2.3. Statistical Analysis

Statistical analyses were performed using StatPlus software (StatPlus Pro v7.3, AnalystSoft, Brandon, FL, USA). Continuous variables were first evaluated for distributional properties using the Shapiro–Wilk test, and homogeneity of variances was assessed with Levene’s test. Quantitative data are presented as mean ± standard deviation (SD).

Differences in elemental concentrations across multiple groups, including histological grades in the endometrial cancer cohort (C, G1, G2, G3), age categories (<50, 50–60, >60 years), and body mass index (BMI) groups (normal weight, overweight, obese), were analyzed using one-way analysis of variance (ANOVA). When statistically significant differences were detected, post hoc comparisons were performed using Tukey’s honestly significant difference (HSD) test to identify specific group differences.

Comparisons between two-group clinical variables, were conducted using the independent samples Student’s *t*-test.

To assess associations between clinical variables and tissue elemental concentrations, separate linear regression models were constructed for each analyte, with elemental concentration as the dependent variable. In univariate analyses, each predictor was entered independently according to the following model: element concentration = β_0_ + β_1_ × predictor + ε. In multivariate analyses, all predictors were entered simultaneously using the following model: element concentration = β_0_ + β_1_ × grade + β_2_ × age + β_3_ × BMI + β_4_ × menopausal status + β_5_ × type 2 diabetes + β_6_ × smoking status + ε. Because age and BMI were available in grouped form in the analytical dataset, these variables were treated as ordered categorical variables in regression models. Menopausal status, type 2 diabetes, and smoking status were included as binary variables.

Regression results are presented as standardized regression coefficients (β), corresponding *p*-values, and coefficients of determination (R^2^). For multivariate models, unstandardized regression coefficients (B) with 95% confidence intervals (95% CI) were additionally calculated to facilitate interpretation of effect size and precision.

Model assumptions, including normality of residuals, linearity, and homoscedasticity, were evaluated by visual inspection of residual plots. Multicollinearity among predictors was assessed using variance inflation factors (VIF), with no evidence of problematic collinearity observed. Correlation analyses between elemental concentrations were conducted using Pearson’s correlation coefficient (r). These analyses were based on individual-level data, with all available samples within each cohort pooled (endometrial cancer cohort: *n* = 50; ovarian cancer cohort: *n* = 41). Prior to correlation analysis, assumptions of approximate normality and linear relationships were evaluated using the Shapiro–Wilk test. No substantial deviations from these assumptions were observed. All statistical tests were two-tailed, and a *p*-value < 0.05 was considered statistically significant.

## 3. Results

### 3.1. Results of Element Concentrations in Endometrioid Endometrial Cancer Tissues and Control Tissues

A progressive alteration in elemental composition was observed across the analyzed groups (C, G1, G2, G3), with several elements demonstrating statistically significant trends.

Among essential macroelements, Na and K concentrations showed a consistent and significant decrease from the control group to G3 (Na: 1840.00 ± 44.18 vs. 1647.14 ± 42.35 µmol/kg, *p* = 0.004; K: 518.62 ± 11.26 vs. 478.86 ± 10.59 µmol/kg, *p* = 0.006). Similarly, Mg and Mn levels decreased progressively across groups (Mg: *p* = 0.010; Mn: *p* = 0.012), as did Cu (*p* = 0.015).

In contrast, Ca concentrations exhibited a significant increasing trend, reaching the highest levels in G3 (114.97 ± 6.64 µmol/kg vs. 95.02 ± 3.58 µmol/kg in controls, *p* = 0.002). Phosphorus (P) demonstrated a modest but statistically significant decline across groups (*p* = 0.049).

Notably, Zn levels did not differ significantly between groups (*p* = 0.210), despite a slight downward tendency, and were therefore not considered differentially expressed.

Among toxic or trace elements, Cr showed a significant decrease across groups (*p* = 0.034), while Be and As exhibited statistically significant but minimal absolute variation (*p* = 0.009 and *p* = 0.041, respectively), suggesting limited biological relevance despite statistical significance.

For several elements, including Mo, Ti, Tl, and Pb, no statistically significant differences were observed (*p*-values not significant), although Pb and Tl displayed a slight increasing trend, whereas Mo showed a gradual decrease across groups. All values and statistical comparisons are presented in Table 3.

To enhance data transparency and account for the wide range of concentration values, individual-level boxplots were included in the Supplementary Materials. For the endometrial cancer cohort (C, G1, G2, G3), macronutrient elements (Na, K, Ca, P, Mg) are presented in Supplementary Figure S1, while trace and toxic elements (Mn, Cu, Zn, Cr, Mo, Ti, Tl, Pb, Be, As) are shown in Supplementary Figure S2.

### 3.2. Age-Related Variations in Tissue Elemental Composition in Endometrial Cancer Tissue

Elemental analysis revealed distinct patterns in mineral composition across study groups and age categories.

In the control group (C), Na concentrations remained relatively stable across age groups, with slightly higher values observed in individuals aged 50–60 and >60 compared to those <50 years. K and P levels showed minimal variation with age. In contrast, Ca demonstrated a gradual increase with age, with significantly higher values observed in individuals > 60 compared to <50. Mg and Mn levels remained relatively consistent across age categories.

In the G1 group, a trend toward lower Na levels compared to C was observed, particularly in individuals < 50. Ca levels were elevated in younger individuals (<50) and showed a decreasing tendency with age. Mg concentrations were significantly lower in the <50 group compared to older subgroups, indicating age-dependent alterations. Trace elements such as Cu and Zn were consistently lower in G1 compared to C, regardless of age.

In the G2 group, Na levels were generally lower compared to both C and G1 across all age categories. Ca concentrations were higher in individuals > 60, with significant differences observed between the 50–60 and >60 subgroups. Mg levels remained relatively stable, while Mn showed moderate variability. Due to the limited sample size in the <50 subgroup (*n* = 1), this category was excluded from inferential statistical analysis; therefore, statistical comparisons were performed only between the 50–60 and >60 subgroups.

In the G3 group, the lowest Na levels among all groups were observed, particularly in older individuals. Ca concentrations were markedly elevated compared to other groups, especially in individuals > 60. P levels showed a decreasing trend with age, while Mg and Mn remained relatively stable. Trace elements such as Cr and Mo tended to be lower in G3 compared to other groups.

Across all groups, Pb and Tl did not show substantial age-dependent variation, although slightly higher Pb levels were observed in G2 and G3 compared to C. As concentrations remained low and relatively stable across all groups and age categories. These age-related differences are shown in Table 4.

### 3.3. BMI-Related Differences in Endometrial Cancer Tissue Elemental Composition

Stratification of elemental concentrations according to BMI revealed relatively limited but element-specific differences within individual clinical groups.

In the control group (C), most elements did not show significant variation across BMI categories. However, Na levels differed significantly between normal and overweight individuals (*p* < 0.05), with lower concentrations observed in the overweight group. Similarly, Ca levels were significantly higher in individuals with normal BMI compared to overweight subjects. Other macroelements, including K, P, and Mg, as well as trace elements (Cu, Zn, Mn, Cr, Mo), remained stable across BMI categories. Pb levels showed a decreasing tendency in obese individuals, although this reached statistical significance only in selected pairwise comparisons.

In the G1 group, BMI-related differences were modest. Na concentrations were significantly lower in obese individuals compared to those with normal BMI (*p* < 0.05), while K levels differed between overweight and obese subgroups. No significant BMI-dependent differences were observed for Ca, P, Mg, or Mn. Similarly, trace elements (Cu, Zn, Be, As, Cr, Mo, Ti, Tl, Pb) remained relatively unchanged across BMI categories, indicating limited metabolic influence of BMI within this group.

In the G2 group, variability was observed for selected elements; however, the pattern differed from that described in other groups. Significant differences were detected primarily between normal and overweight subgroups, including Na, K, Ca, and P (*p* < 0.05), as indicated in Table 5. No consistent differences were observed between overweight and obese individuals. Due to the small sample size in the normal BMI subgroup (*n* = 2), these findings should be interpreted with caution.

In the G3 group, the normal BMI subgroup (*n* = 1) was excluded from inferential statistical analysis. Therefore, comparisons were performed only between overweight and obese individuals using Student’s *t*-test. Significant differences were observed between these two subgroups for selected macroelements, including Ca and P (*p* < 0.05), with the overweight group showing higher Ca and lower P levels compared to obese individuals. Na also demonstrated variability between subgroups, although no consistent pattern was observed. Other elements, including Mg, Mn, and trace metals, did not show significant BMI-dependent differences.

Across all groups, the majority of trace elements (Cu, Zn, Be, As, Cr, Mo, Ti, Tl) remained relatively stable irrespective of BMI. All BMI-related findings are summarized in Table 5.

### 3.4. Menopause-Related Variations in Elemental Composition in Endometrial Cancer Tissue

Elemental concentrations stratified by menopausal status demonstrated generally limited differences between premenopausal (No) and postmenopausal (Yes) patients across the analyzed clinical groups.

In the control group (C), postmenopausal women exhibited significantly higher levels of Na and K compared to premenopausal individuals (*p* < 0.05). In contrast, Ca, P, Mg, and Mn levels remained comparable between subgroups. Trace elements, including Cu, Zn, Be, As, Cr, Mo, Ti, Tl, and Pb, showed minimal variation, indicating that menopausal status had a limited impact on elemental distribution in the control cohort.

In the G1 group, statistically significant differences were observed only for K, with higher concentrations in postmenopausal individuals compared to premenopausal patients (*p* < 0.05). Other macroelements (Na, Ca, P, Mg) and trace elements (Cu, Zn, Be, As, Cr, Mo, Ti, Tl, Pb) remained stable across menopausal status, suggesting limited influence of hormonal changes on elemental profiles in this group.

In the G2 group, statistical comparisons between menopausal subgroups were not performed due to the insufficient number of premenopausal patients (*n* = 1). Although descriptive differences were observed, including higher Na and P and lower Ca levels in postmenopausal individuals, these findings should be interpreted with caution and were not subjected to inferential statistical analysis.

The G3 group was excluded from menopausal status comparisons, as all patients in this subgroup were postmenopausal, precluding any comparative analysis. The complete results for menopausal status are detailed in Table 6.

### 3.5. Influence of Type 2 Diabetes on Elemental Profiles in Endometrial Cancer Tissue

Elemental concentrations stratified according to diabetes status revealed generally limited but statistically significant differences for selected elements within individual clinical groups.

In the control group (C), Na levels differed significantly between diabetic and non-diabetic individuals (*p* < 0.05), with higher values observed in the diabetic subgroup. Despite this difference, other macroelements, including K, Ca, P, Mg, and Mn, remained comparable between subgroups. Similarly, trace elements (Cu, Zn, Be, As, Cr, Mo, Ti, Tl, Pb) did not show significant variation, indicating a minimal overall impact of diabetes on elemental homeostasis in the control cohort.

In the G1 group, a statistically significant difference was observed for Na (*p* < 0.05), with lower concentrations in diabetic patients compared to non-diabetic individuals. No significant differences were detected for K, Ca, P, Mg, or Mn. Additionally, trace elements, including Cu, Zn, Be, As, Cr, Mo, Ti, Tl, and Pb, remained stable across diabetes status, suggesting limited metabolic influence of diabetes within this group.

In the G2 group, Na levels were significantly lower in diabetic individuals compared to non-diabetic subjects (*p* < 0.05). Although slight variations were observed for Ca and P, these differences were not statistically significant. Other analyzed elements, including Mg, Mn, and trace metals, showed no meaningful differences between subgroups.

In the G3 group, Na concentrations were significantly higher in diabetic patients compared to non-diabetic individuals (*p* < 0.05). However, other macroelements (Ca, P, Mg) and trace elements (Cu, Zn, Be, As, Cr, Mo, Ti, Tl, Pb) did not differ significantly between subgroups, and no consistent diabetes-dependent pattern was observed. All diabetes-related comparisons are presented in Table 7.

### 3.6. Influence of Cigarette Smoking on Elemental Composition in Endometrium Cancer Cohort

Elemental concentrations stratified according to smoking status revealed generally limited differences between smokers and non-smokers, with statistically significant differences observed primarily for Na and K in selected groups.

In the control group (C), smokers exhibited significantly lower Na and K concentrations compared to non-smokers (*p* < 0.05). In contrast, other macroelements, including Ca, P, Mg, and Mn, remained comparable between subgroups. Similarly, trace elements (Cu, Zn, Be, As, Cr, Mo, Ti, Tl, Pb) showed no significant differences, indicating that smoking had a selective rather than global effect on elemental composition in the control group.

In the G1 group, a statistically significant difference was observed for Na (*p* < 0.05), with slightly lower concentrations in smokers compared to non-smokers. No significant differences were detected for K, Ca, P, Mg, or Mn. Additionally, trace elements remained stable across smoking status, suggesting minimal influence of smoking on elemental homeostasis in this group.

In the G2 group, no statistically significant differences were observed between smokers and non-smokers for any of the analyzed elements. Although minor variations were noted for Ca and P, these differences were not significant, and trace element concentrations remained largely unchanged.

In the G3 group, smokers exhibited significantly lower Na levels compared to non-smokers (*p* < 0.05). However, other macroelements (Ca, P, Mg) and trace elements did not differ significantly between subgroups, and no consistent smoking-related pattern was observed.

Overall, smoking status was associated with statistically significant differences primarily in Na concentrations across multiple groups and in K within the control group. All smoking related comparisons are presented in Table 8.

### 3.7. Correlation Analysis of Macro- and Microelements in Endometrial Cancer Tissue

Correlation analysis demonstrated multiple statistically significant associations between the analyzed elements (Table 9).

Significant positive correlations (*p* < 0.05) were identified among Na, K, Mg, Mn, Cu, Zn, and Cr. The strongest correlations were observed for Na–Cr (r = 0.77), Na–Mn (r = 0.75), Na–K (r = 0.71), and K–Cr (r = 0.68). Additional significant positive relationships included Mg–Cu (r = 0.63), Cu–Zn (r = 0.62), and Mg–Mn (r = 0.61).

Significant negative correlations were noted between Ca and multiple elements, including K (r = −0.66), Cr (r = −0.63), and Na (r = −0.61). Negative associations were also observed between Ca and Mg, Mn, Cu, and Zn (all *p* < 0.05).

P showed significant positive correlations with Na (r = 0.55), K (r = 0.52), Mg (r = 0.36), and Mn (r = 0.45), as well as a negative correlation with Ca (r = −0.55).

Among trace elements, Ti demonstrated significant positive correlations with Ca (r = 0.48), Tl (r = 0.31), and Pb (r = 0.25), and negative correlations with Na, Mn, and Cr (all *p* < 0.05). A positive correlation was also observed between Tl and Pb (r = 0.32, *p* < 0.05).

As showed significant negative correlations with Na (r = −0.37), K (r = −0.29), Mg (r = −0.32), Mn (r = −0.36), Cu (r = −0.33), Zn (r = −0.31), and Cr (r = −0.35), as well as a positive correlation with Ca (r = 0.29). Be demonstrated limited associations, with only a weak positive correlation with P (r = 0.26, *p* < 0.05).

### 3.8. Regression Analyses of Elemental Profiles in Endometrioid Endometrial Cancer Tissue

Regression analyses were performed to identify clinical and lifestyle factors associated with tissue elemental composition in the endometrial cancer cohort. Univariate models included each predictor separately, whereas multivariate models simultaneously incorporated histological grade, age, BMI, menopausal status, type 2 diabetes, and smoking status.

In univariate analyses (Table 10), histological grade emerged as the most consistent determinant across the majority of analytes, showing strong inverse associations with Na, K, Mg, Mn, Cu, Zn, and Cr, and positive associations with Ca, Ti, Tl, and Pb. By contrast, age and BMI demonstrated more limited and element-specific effects. Age was associated mainly with increased Ca and Ti levels, whereas BMI reached significance only for selected analytes, including Na and Mn. Smoking status was not significantly associated with elemental concentrations in the univariate models.

Multivariate regression analyses incorporating histological grade, age, BMI, menopausal status, type 2 diabetes, and smoking status demonstrated that histological grade was the strongest and most consistent independent predictor of elemental composition in endometrial cancer tissue (Table 11). Increasing tumor grade was associated with significant decreases in Na (B = −62.69, 95% CI: −77.06 to −48.32), K (B = −12.71, 95% CI: −17.24 to −8.18), P (B = −4.16, 95% CI: −6.46 to −1.86), Mg (B = −1.77, 95% CI: −2.50 to −1.03), Mn (B = −4.02, 95% CI: −5.02 to −3.02), Cu (B = −0.140, 95% CI: −0.209 to −0.071), and Cr (B = −2.65, 95% CI: −3.48 to −1.82). Conversely, positive associations with tumor grade were observed for Ca (B = 7.41, 95% CI: 4.87 to 9.96), Ti (B = 2.04, 95% CI: 1.53 to 2.55), Tl (B = 0.064, 95% CI: 0.033 to 0.096), and Pb (B = 0.158, 95% CI: 0.068 to 0.247). These associations were characterized by relatively narrow confidence intervals, supporting the robustness of the grade-dependent shifts in elemental homeostasis.

Among the remaining predictors, age showed a limited and element-specific effect, reaching borderline significance only for Mg (B = 0.77, 95% CI: 0.00 to 1.54), while confidence intervals for most other elements crossed zero. BMI did not demonstrate independent associations with elemental concentrations after adjustment, with all confidence intervals including zero.

Menopausal status was significantly associated only with selected elements, most notably Mn (B = 2.25, 95% CI: 0.03 to 4.47) and Cr (B = 2.46, 95% CI: 0.62 to 4.30), suggesting a potential hormonal contribution to trace element regulation. Other variables, including type 2 diabetes, showed no consistent independent effects across analytes.

Interestingly, smoking status exhibited a selective association with Cu (B = 0.118, 95% CI: 0.015 to 0.220) and a weak inverse association with As (B = −0.0014, 95% CI: −0.0027 to −0.0001), while remaining non-significant for most elements.

The explanatory power of the models varied considerably, with R^2^ values ranging from 0.04 (Be) to 0.73 (Na), indicating substantial heterogeneity in the extent to which elemental variability was explained by the included clinical parameters. The highest model fits were observed for Na, Ti, Mn, and Cr, further underscoring the dominant role of tumor grade in shaping elemental profiles.

### 3.9. Results of Element Concentrations in Ovarian Cancer Tissue and Control Tissue

Elemental analysis across ovarian cancer subgroups (Group A, Group B, Group C) revealed several statistically significant differences, although the magnitude and consistency of these changes varied between elements.

Among essential macroelements, Na and K concentrations showed a gradual increase from Group A to Group C (Na: 1750.00 ± 42.00 vs. 1820.00 ± 45.00 µmol/kg, *p* = 0.038; K: 495.00 ± 11.00 vs. 510.00 ± 12.00 µmol/kg, *p* = 0.041). Similarly, Mn and Cu levels increased significantly across groups (Mn: *p* = 0.031; Cu: *p* = 0.039), suggesting a trend toward higher concentrations in less advanced or differently treated cases.

In contrast, Ca demonstrated a decreasing pattern, with the highest levels observed in Group A and the lowest in Group C (*p* = 0.014). Ti also showed a statistically significant decrease across groups (*p* = 0.028).

Phosphorus (P) exhibited an increasing tendency; however, no statistically significant difference was reported. Likewise, Mg and Zn showed upward trends, but these did not reach statistical significance (*p* = 0.071 and *p* = 0.083, respectively).

Among trace and toxic elements, no significant differences were observed for Cr, Tl, or Pb, although Pb demonstrated a slight decreasing tendency across groups. Similarly, Be, As, and Mo showed minimal variation without statistical significance, indicating relative stability across ovarian cancer subgroups. These findings are presented in Table 12.

To enhance data transparency and account for the wide range of concentration values, individual-level boxplots were included in the Supplementary Materials. For the ovarian cancer cohort (A, B, C), macronutrient elements (Na, K, Ca, P, Mg) are presented in Supplementary Figure S3, while trace and toxic elements (Mn, Cu, Zn, Cr, Mo, Ti, Tl, Pb, Be, As) are shown in Supplementary Figure S4.

### 3.10. Age-Related Variations in Tissue Elemental Composition in Ovarian Cancer Tissue

Across all groups, age-related trends were consistent and more pronounced than intergroup differences.

Na and K decreased with age in all groups. The highest values were observed in the <50 subgroup, while the lowest occurred in >60, with several significant differences, particularly between <50 and >60 (e.g., K in A and C).

Ca and Mg also declined progressively with age across A, B, and C. These changes were statistically significant in multiple comparisons, especially between younger (<50) and older (>60) subgroups.

P showed a mild downward trend with age, but without clear statistical significance.

Mn and Cu followed a decreasing pattern with advancing age in all groups, although changes were modest. Zn similarly declined, with significant differences mainly between <50 and >60 subgroups.

Cr also decreased with age, reaching significance in several comparisons (notably in A and B). Mo showed a slight reduction, though without consistent significance.

In contrast, As and Tl exhibited a slight increase with age, although these changes were small and not statistically significant. Pb tended to increase with age, with some significant differences observed (e.g., in B and C between younger and older subgroups).

Ti did not follow a consistent age-related pattern and remained relatively stable across subgroups.

Overall, younger patients (<50) were characterized by higher Na, K, Ca, Mg, Zn, and Cr levels, whereas older individuals (>60) showed reduced concentrations of these elements and a tendency toward higher Pb. These results are shown in Table 13.

### 3.11. BMI-Related Differences in Ovarian Tissue Elemental Composition

Across all groups, BMI-related differences showed a consistent pattern.

Na and K decreased with increasing BMI in A, B, and C. The highest values were observed in normal BMI, while obese patients exhibited the lowest levels, with several significant differences (particularly normal vs. obese).

Ca, P, and Mg followed a similar downward trend with increasing BMI. These reductions were most evident in obese subgroups and reached statistical significance in multiple comparisons.

Mn and Zn also decreased with higher BMI, with the lowest values consistently observed in obese individuals. Cu showed a mild decline, although changes were less pronounced.

Cr demonstrated a gradual reduction with increasing BMI, while Mo showed a slight decrease without consistent significance.

In contrast, As and Tl tended to increase with BMI, though these changes were small and not statistically significant. Pb showed a consistent increase from normal to obese across all groups, with higher levels in obese patients.

Ti remained relatively stable, with no clear BMI-dependent trend.

Overall, normal BMI was associated with higher Na, K, Ca, Mg, Mn, and Zn, whereas obesity was linked to reduced concentrations of these elements and a tendency toward increased Pb. The complete results are presented in Table 14.

### 3.12. Menopause-Related Variations in Elemental Composition in Ovarian Cancer Tissue

Menopause status was associated with clear and consistent differences across all groups.

Na and K were higher in premenopausal patients (No) compared to postmenopausal (Yes) in A, B, and C, with multiple significant differences. A similar pattern was observed for Ca, P, and Mg, all showing reduced levels after menopause.

Mn, Cu, and Zn were also consistently lower in postmenopausal individuals, with several statistically significant differences, particularly in Groups A and C.

Cr and Mo decreased after menopause, with significant differences noted mainly in A and C. Ti showed a slight increase in postmenopausal patients, though without a consistent pattern.

In contrast, As and Tl tended to be higher in postmenopausal groups, although these changes were generally not significant.

Pb showed an increasing trend after menopause, with higher values in postmenopausal patients, reaching significance in some comparisons (notably in A and C).

Overall, premenopausal status was associated with higher Na, K, Ca, Mg, Mn, Cu, Zn, Cr, and Mo, whereas postmenopausal patients exhibited reduced levels of these elements and a tendency toward higher Pb. These results are detailed in Table 15.

### 3.13. Influence of Type 2 Diabetes on Elemental Profiles in Ovarian Tissue

Diabetes status was associated with consistent and directionally similar changes across all groups.

Na and K were lower in diabetic patients (Yes) compared to non-diabetic (No) in A, B, and C, with statistically significant differences. Mg showed a similar decrease in diabetic individuals across all groups.

In contrast, Ca was higher in diabetic patients in all groups, showing a consistent opposite trend compared to Na and K. P generally decreased in diabetes, particularly in A and C.

Cu and Zn were reduced in diabetic patients, with significant differences observed across most groups. Cr and Mo also showed lower values in the diabetic subgroup, although changes were less pronounced.

Mn did not show a consistent pattern and remained relatively stable between diabetic and non-diabetic patients.

Ti and Tl tended to be higher in diabetic individuals, with several significant differences, especially in A and C.

Pb showed a clear increase in diabetic patients across all groups, with consistently higher values compared to non-diabetic individuals.

Overall, diabetes was associated with lower Na, K, Mg, Cu, Zn, Cr, and Mo, alongside higher Ca, Ti, Tl, and Pb, while Mn remained relatively unchanged. All results are presented in Table 16.

### 3.14. Influence of Cigarette Smoking on Elemental Composition in Ovarian Cancer Cohort

Smoking status showed relatively minor and inconsistent effects compared to other variables.

Na and K did not demonstrate a clear or consistent pattern between smokers and non-smokers across groups. Differences were small and generally not significant.

Ca showed opposite tendencies depending on the group: in B, higher values were observed in smokers, whereas in C a slight decrease was noted. In A, values remained nearly unchanged.

P and Mg exhibited minimal variation between smokers and non-smokers, without a consistent directional trend.

Mn and Cu also showed only subtle differences, with no clear smoking-related pattern.

Zn tended to be slightly higher in smokers, particularly in C, where the difference reached significance.

Cr showed a decrease in smokers in B, with significant differences, while remaining relatively stable in A and C.

Mo and Ti did not demonstrate meaningful variation with smoking status.

Be, As, Tl, and Pb remained largely unchanged between smokers and non-smokers across all groups.

Overall, smoking had a limited impact on elemental concentrations, with only isolated differences (notably Zn and Cr), and no consistent systemic trend across groups. All results are presented in Table 17.

### 3.15. Correlation Analysis of Macro- and Microelements in Ovarian Cancer Tissue

Correlation analysis revealed two clearly opposing clusters of elements.

Na, K, Mg, Zn, Cu, Cr, Mo, and P formed a strong positively correlated group. The strongest relationships were observed between Zn and Ca (negative, r = −0.95), Mg and Ca (r = −0.92), and Na with Zn (r = 0.83). Within this cluster, Zn, Mg, and Na showed particularly strong positive intercorrelations, indicating a tightly linked metabolic profile.

Ca exhibited strong negative correlations with most elements from this group, including Mg (r = −0.92), Zn (r = −0.95), Na (r = −0.79), and K (r = −0.75), suggesting an inverse regulatory relationship.

Pb, Tl, and Ti showed a pattern similar to Ca, with predominantly negative correlations with Na, K, Mg, and Zn, and positive associations with Ca (e.g., Pb–Ca: r = 0.67). Pb demonstrated strong negative correlations with Zn (r = −0.70) and Na (r = −0.69).

As behaved oppositely to the main cluster, showing positive correlations with Ca (r = 0.54) and Pb (r = 0.47), and negative correlations with Zn (r = −0.52), Cu (r = −0.56), and Mg (r = −0.47).

Mn showed weaker and less consistent relationships, with moderate positive correlations with Cr (r = 0.56) and Zn (r = 0.48), but generally low association with other elements.

Be demonstrated mostly weak correlations, with only modest positive relationships with Mg (r = 0.43) and Zn (r = 0.38).

Overall, the data indicate a dominant axis characterized by positive intercorrelations among Na–K–Mg–Zn–Cu–Cr–Mo–P, opposed by Ca and Pb (and partially Tl and Ti), suggesting two biologically distinct elemental profiles. All results are presented in Table 18.

### 3.16. Regression Analyses of Elemental Profiles in Ovarian Cancer Tissue

Univariate regression analysis demonstrated strong associations between treatment and elemental concentrations, along with notable effects of BMI and selected age-related influences.

Treatment (B vs. A) showed significant negative associations with Na, K, P, Mg, Mn, Cu, Zn, Cr, and Mo, indicating reduced levels in Group B. In contrast, Ca, As, Ti, Tl, and Pb were positively associated with treatment, with the strongest effects observed for Ca (β = +0.68) and Ti (β = +0.51).

Age showed a pronounced negative association with Zn (β = −0.85), representing the strongest age-related effect. Additionally, Ca (β = +0.27) and Ti (β = +0.38) increased with age, while Na (β = −0.21) and Mo (β = −0.29) decreased.

BMI was strongly associated with multiple elements. Negative relationships were observed for Na (β = −0.74), Mg (β = −0.82), K (β = −0.42), Mn (β = −0.35), and Cu (β = −0.39). In contrast, Ca showed a very strong positive association with BMI (β = +0.94), representing the most pronounced effect in the model.

Menopause, diabetes, and smoking did not demonstrate meaningful associations with most elements, with only minor trends observed.

Overall, treatment and BMI emerged as the dominant factors influencing elemental composition, with age exerting selective effects, particularly on Zn, Ca, Ti, Na, and Mo (Table 19).

Multivariate regression analyses adjusted for group, age, BMI, menopausal status, type 2 diabetes, and smoking status demonstrated that group classification was the primary determinant of elemental composition in ovarian cancer tissue (Table 20).

Group status was significantly associated with reduced concentrations of Na (B = −47.55, 95% CI: −64.41 to −30.68), K (B = −11.28, 95% CI: −15.89 to −6.68), P (B = −4.01, 95% CI: −5.74 to −2.28), Mg (B = −1.70, 95% CI: −2.13 to −1.27), Mn (B = −2.76, 95% CI: −3.52 to −1.99), Cu (B = −0.18, 95% CI: −0.24 to −0.12), Zn (B = −0.12, 95% CI: −0.15 to −0.10), Cr (B = −2.83, 95% CI: −3.57 to −2.08), and Mo (B = −0.08, 95% CI: −0.12 to −0.04). In contrast, positive associations were observed for Ca (B = 5.76, 95% CI: 4.83 to 6.68), Ti (B = 0.58, 95% CI: 0.01 to 1.15), Tl (B = 0.05, 95% CI: 0.01 to 0.08), and Pb (B = 0.17, 95% CI: 0.10 to 0.24). These findings indicate a consistent group-dependent shift in elemental homeostasis, with relatively narrow confidence intervals supporting the robustness of these effects.

Among the remaining predictors, age and BMI showed minimal independent influence, with confidence intervals for most elements including zero, indicating a lack of statistically significant associations after adjustment.

Menopausal status demonstrated selective associations, particularly with Mn (B = 1.13, 95% CI: 0.03 to 2.23) and Cr (B = 1.31, 95% CI: 0.32 to 2.29), suggesting a potential hormonal contribution to trace element variability.

Type 2 diabetes exhibited element-specific effects, including positive associations with Mn (B = 3.47, 95% CI: 2.27 to 4.67), Cr (B = 1.53, 95% CI: 0.37 to 2.69), Ca (B = 6.26, 95% CI: 4.81 to 7.70), and Pb (B = 0.16, 95% CI: 0.04 to 0.27), as well as an inverse association with Mg (B = −1.56, 95% CI: −2.23 to −0.89).

Smoking status did not show consistent independent associations, with most confidence intervals crossing zero.

The explanatory power of the models varied across analytes, with R^2^ values ranging from 0.23 (Be) to 0.92 (Ca). The highest model fits were observed for Ca, Mg, and Zn, indicating that the included clinical variables—particularly group status—substantially explained variability in these elemental concentrations.

## 4. Discussion

The present study provides a comprehensive evaluation of macro- and trace element profiles in endometrial cancer, demonstrating that elemental alterations are predominantly associated with tumor progression rather than systemic metabolic or lifestyle-related factors. The observed increase in Ca, P, Mg, and Mn in higher-grade tumors may reflect enhanced tumor metabolic activity and proliferation, as these elements are involved in key cellular and enzymatic processes. In contrast, most trace elements, including Zn, Ti, Tl, and Pb, did not show significant differences across tumor grades, suggesting a limited role in endometrial cancer progression. Stratified analyses further demonstrated that clinical and metabolic factors, such as age, BMI, menopausal status, diabetes, and smoking, had only minor and inconsistent effects on elemental concentrations, without a clear biological pattern. Multivariate analysis confirmed histological grade as the primary determinant of elemental variability, while other factors contributed only marginally, highlighting the dominant influence of tumor-driven mechanisms.

The observed decrease in Na and K concentrations across tumor grades, accompanied by strong positive correlations between these ions, suggests coordinated alterations in ionic homeostasis. Na^+^/K^+^-ATPase activity has been implicated in processes such as proliferation, apoptosis, and epithelial–mesenchymal transition [40]. However, in the present study, reduced Na and K levels are more likely to reflect altered tissue architecture, hypoxia, cellular density, and disrupted transmembrane transport, rather than direct mechanistic drivers of malignant transformation [41,42]. The additional associations with age and BMI further suggest that systemic metabolic status may be mirrored at the tissue level, although these relationships remain observational and potentially confounded [43].

In contrast, Ca concentrations increased with disease advancement and adverse metabolic profiles. Given the established role of Ca^2+^ as a second messenger in proliferation, migration, and apoptosis resistance [44,45], These findings are biologically plausible. Nonetheless, elevated Ca levels in tumor tissue may also arise from non-specific processes, including microcalcification, stromal remodeling, necrosis, or altered extracellular matrix composition, rather than direct functional involvement in tumor progression [46,47,48,49]. Therefore, Ca enrichment should be interpreted cautiously as a potential feature of the tumor microenvironment rather than a direct pathogenic factor. Phosphorus exhibited relatively stable levels across groups, despite its central role in cellular energetics and membrane structure. This relative stability may reflect tight homeostatic regulation required to sustain proliferative activity, while its correlations with other elements suggest participation in broader metabolic networks rather than independent behavior [50,51,52,53].

Among essential trace elements, Mg showed one of the most consistent reductions in advanced disease and in patients with adverse metabolic characteristics. Given its role in ATP-dependent reactions, DNA repair, and enzymatic function, decreased Mg levels may reflect increased metabolic demand, intracellular redistribution, or altered transport within tumor tissues, rather than systemic deficiency per se [54,55].

Similarly, reductions in Zn and Cu may be associated with disruptions in redox balance and antioxidant defense systems, including Cu/Zn superoxide dismutase activity [56,57].

However, these interpretations are based on established biological functions and should not be considered direct evidence of mechanistic involvement in the present cohort [58,59,60,61,62,63,64,65,66,67].

Cr concentrations were also lower in more advanced disease and in metabolically burdened patients. As Cr is involved in glucose metabolism and insulin signaling [68,69], its reduction may be associated with broader metabolic dysregulation. Notably, its correlations with other elements suggest that Cr alterations occur as part of a coordinated metabolic network, rather than as an isolated phenomenon [70].

Mo showed relatively limited variation across groups, suggesting a more stable role within enzymatic systems. Its correlations with other elements may indicate involvement in shared redox pathways, although its discriminatory value in this context appears limited [34]. Its relatively stable levels indicate limited discriminatory value in this cohort.

Among toxic elements, Pb and Tl demonstrated the most consistent increases with tumor progression and adverse clinical features. Pb has been described as a metalloestrogen and a potential inducer of oxidative stress [39,71,72]; however, the present findings do not allow determination of its role in tumor initiation or progression. Instead, its accumulation may reflect altered tissue retention, environmental exposure, or changes in vascularization and stromal composition. Tl exhibited a similar pattern, although its biological relevance in cancer remains less well defined. Ti increases may also reflect environmental exposure or deposition in structurally altered tissue [73].

In contrast, Be and As showed minimal variation, suggesting limited relevance within the analyzed disease stages or cohort [17,74].

Other important observation is that elemental alterations were more strongly associated with tumor-related variables than with systemic clinical factors. While age and BMI showed some associations, menopause and diabetes exerted relatively modest and inconsistent effects. This suggests that the observed elemental patterns are more closely linked to tumor biology and microenvironmental conditions than to systemic exposures alone, although residual confounding cannot be excluded [75,76,77].

Correlation analyses further demonstrated that elemental changes occur in coordinated clusters rather than independently. This supports the concept that elemental dyshomeostasis represents an integrated network-level phenomenon, reflecting alterations in transport systems, metabolic pathways, and the tumor microenvironment, rather than isolated disturbances in individual metals [17,39,78].

Several important limitations should be considered when interpreting the results of this study.

First, the cross-sectional and tissue-based design precludes any inference regarding causality or temporal relationships. The observed associations cannot distinguish whether elemental alterations contribute to tumor development or arise as a consequence of tumor-related metabolic and microenvironmental changes.

Second, the sample size was relatively modest, particularly in specific subgroups, including patients with G3 endometrial cancer and those with ovarian cancer treated with surgery alone. These smaller subgroup sizes may limit statistical power and increase the risk of both type I and type II errors.

Third, no correction for multiple testing was applied despite the large number of analyzed elements and comparisons. This increases the likelihood of false-positive findings and necessitates cautious interpretation of statistically significant results.

Fourth, although efforts were made to standardize sample handling, pre-analytical variability cannot be fully excluded. Factors such as tissue collection, processing, storage, and mineralization may influence elemental concentrations. In addition, while analytical quality control measures were implemented, detailed assay validation specific to all analyzed elements in this exact tissue matrix remains limited, which may affect measurement accuracy.

Fifth, the use of ICP-OES, while well-established, is subject to potential matrix effects and contamination risks, particularly in trace element analysis. Despite strict contamination-control procedures, exogenous contamination or matrix-related signal interference cannot be entirely ruled out.

Sixth, the ovarian cancer cohort was heterogeneous, and grouping based on treatment strategy does not fully account for differences in tumor stage, histological subtype, or molecular classification. The lack of stage-adjusted and histotype-adjusted analyses represents an important limitation that may confound interpretation of the observed associations.

Seventh, although multiple clinical variables were included, residual confounding remains possible, particularly with respect to environmental exposure, dietary factors, and unmeasured metabolic variables.

Finally, the study lacks external validation in independent cohorts, which limits the generalizability of the findings. Replication in larger, well-characterized populations and integration with longitudinal and functional studies are necessary to confirm the robustness and biological relevance of the observed elemental patterns.

Taken together, the identified pattern—characterized by relatively higher Ca and Pb and lower Mg, Zn, Cu, and Cr—may represent a composite tissue signature associated with tumor presence and progression. However, its biological and clinical significance remains to be established. The similarities observed between endometrial and ovarian cancers suggest shared metabolic or microenvironmental features, although differences in magnitude may reflect underlying epidemiological and metabolic distinctions, particularly the stronger association of endometrial cancer with obesity and metabolic dysfunction.

From a clinical perspective, these findings should be considered exploratory. While selected elements may serve as potential tissue-level indicators associated with disease characteristics, the current data are insufficient to support their use as diagnostic biomarkers or therapeutic targets. Further validation in independent cohorts, as well as integration with molecular and functional analyses, is required to determine their clinical relevance.

## 5. Conclusions

This study demonstrates that elemental homeostasis in endometrial cancer is primarily associated with tumor progression rather than systemic metabolic or lifestyle-related factors. Significant alterations in macroelements, particularly Ca, P, Mg, and Mn, were observed across histological grades, suggesting their involvement in tumor-related metabolic reprogramming and microenvironmental remodeling.

In contrast, the majority of trace elements, including Zn, Ti, Tl, and Pb, did not show statistically significant differences across cancer grades, indicating a relatively stable profile independent of tumor differentiation. Similarly, clinical variables such as age, BMI, menopausal status, diabetes, and smoking exerted only limited and inconsistent effects on elemental concentrations, with no clear global pattern identified.

Multivariate analysis further confirmed that histological grade represents the strongest determinant of elemental variability, whereas other clinical and metabolic factors contribute in a secondary and element-specific manner.

Overall, these findings suggest that elemental alterations in endometrial cancer are predominantly driven by tumor biology rather than systemic metabolic disturbances. Although elemental profiling may offer insights into cancer-related metabolic changes, its potential diagnostic or prognostic value requires further validation in larger, well-characterized cohorts.

## Figures and Tables

**Table 1:** Demographic and Clinical Characteristics of the Study Population Across Control and Endometrial Cancer Subgroups.

Variable		C (*n* = 20)	G1 (*n* = 28)	G2 (*n* = 13)	G3 (*n* = 9)
Age	<50 years	3	6	1	0
	50–60 years	9	12	5	3
	>60 years	8	10	7	6
BMI [kg/m^2^]	Normal	8	6	2	1
	Overweight	7	11	4	3
	Obese (<40)	5	11	7	5
Cigarette smoking	Yes	6	9	5	4
	No	14	19	8	5
Menopause status	Yes	16	22	12	9
	No	4	6	1	0
Type 2 diabetes	Yes	3	7	5	4
	No	17	21	8	5

**Table 2:** Demographic and Clinical Characteristics of Patients With Ovarian Cancer and Non-Oncological Controls.

Variable		Group A (Surgery + Chemotherapy; *n* = 28)	Group B (Surgery Only; *n* = 13)	Group C (*n* = 25)
Age	<50 years	3	4	5
	50–60 years	10	5	9
	>60 years	15	4	11
BMI [kg/m^2^]	Normal	6	5	9
	Overweight	11	4	8
	Obese (BMI < 40)	11	4	8
Cigarette smoking	Yes	10	4	7
	No	18	9	18
Menopause status	Yes	23	10	18
	No	5	3	7
Type 2 diabetes	Yes	9	3	4
	No	19	10	21

**Table 3:** Differences in elemental concentrations between the endometrial cancer subgroups and controls.

Element	C	G1	G2	G3	*p* (ANOVA)	Tukey
Na (µmol/kg)	1840.00 ± 44.18	1776.91 ± 36.05	1703.32 ± 24.43	1647.14 ± 42.35	0.004	a, b, c, d, f
K (µmol/kg)	518.62 ± 11.26	503.19 ± 11.19	493.89 ± 9.05	478.86 ± 10.59	0.006	a, b, c, d, f
Ca (µmol/kg)	95.02 ± 3.58	100.52 ± 6.02	105.78 ± 5.84	114.97 ± 6.64	0.002	a, b, c, d, e, f
P (µmol/kg)	136.93 ± 2.72	135.42 ± 4.92	132.87 ± 6.02	126.77 ± 6.28	0.049	a, b, c
Mg (µmol/kg)	48.34 ± 1.53	46.10 ± 1.63	43.59 ± 1.54	43.36 ± 2.24	0.010	a, b, c, d, f
Mn (µmol/kg)	53.84 ± 2.33	51.73 ± 2.25	47.26 ± 2.07	44.92 ± 3.32	0.012	a, b, c, d, f
Cu (µmol/kg)	3.10 ± 0.10	2.80 ± 0.16	2.58 ± 0.21	2.52 ± 0.13	0.015	a, b, c, f
Zn (µmol/kg)	1.89 ± 0.07	1.75 ± 0.06	1.70 ± 0.08	1.70 ± 0.11	0.210	—
Be (µmol/g)	21.51 ± 0.88	21.20 ± 0.67	21.14 ± 1.02	21.07 ± 1.24	0.009	a, b, c, d, f
As (µmol/g)	0.03 ± 0.00	0.03 ± 0.00	0.03 ± 0.00	0.03 ± 0.00	0.041	b, c, d, f
Cr (µmol/g)	50.37 ± 1.23	47.66 ± 1.80	45.32 ± 2.25	42.98 ± 2.67	0.034	e, f
Mo (µmol/g)	1.85 ± 0.05	1.77 ± 0.12	1.71 ± 0.12	1.66 ± 0.09	—	—
Ti (µmol/g)	50.04 ± 1.52	48.91 ± 1.15	51.66 ± 1.00	53.02 ± 1.42	—	—
Tl (µmol/g)	2.12 ± 0.07	2.12 ± 0.08	2.20 ± 0.07	2.22 ± 0.07	—	—
Pb (µmol/g)	4.77 ± 0.18	4.91 ± 0.20	5.03 ± 0.24	5.18 ± 0.27	—	—

Data are presented as mean ± standard deviation (SD). Na, sodium; K, potassium; Ca, calcium; P, phosphorus; Mg, magnesium; Mn, manganese; Cu, copper; Zn, zinc; Be, beryllium; As, arsenic; Cr, chromium; Mo, molybdenum; Ti, titanium; Tl, thallium; Pb, lead. Statistical significance was assessed using one-way ANOVA followed by Tukey’s post hoc test. Significant pairwise comparisons: a, G1 vs. C (*p* < 0.05); b, G2 vs. C (*p* < 0.05); c, G3 vs. C (*p* < 0.05); d, G2 vs. G1 (*p* < 0.05); e, G2 vs. G3 (*p* < 0.05); f, G3 vs. G1 (*p* < 0.05).

**Table 4:** Elemental concentrations stratified by age in the endometrial cancer cohort.

Group	Age (*n*)	Na	K	Ca	P	Mg	Mn	Cu	Zn	Be	As	Cr	Mo	Ti	Tl	Pb
C	<50 (*n* = 3)	1808.84 ± 42.87 ^ab^	518.63 ± 10.82 ^a^	93.20 ± 2.81 ^b^	137.24 ± 2.59	47.72 ± 1.91	53.78 ± 0.59	3.165 ± 0.083	1.946 ± 0.069	20.69 ± 1.25	0.025 ± 0.001	50.86 ± 0.95	1.855 ± 0.067	48.01 ± 1.25	2.101 ± 0.053	4.761 ± 0.261
	50–60 (*n* = 9)	1846.48 ± 48.78 ^c^	516.11 ± 9.22	94.58 ± 3.67	136.86 ± 3.07	48.37 ± 1.71	54.00 ± 2.50	3.078 ± 0.105	1.859 ± 0.073	21.38 ± 0.66	0.025 ± 0.001	50.33 ± 1.52	1.867 ± 0.039	50.03 ± 1.57	2.121 ± 0.082	4.734 ± 0.205
	>60 (*n* = 8)	1844.39 ± 39.51	521.44 ± 14.02 ^c^	96.21 ± 3.71	136.89 ± 2.71	48.54 ± 1.34	53.68 ± 2.74	3.099 ± 0.110	1.898 ± 0.038	21.97 ± 0.78	0.025 ± 0.001	50.23 ± 1.03	1.821 ± 0.057	50.82 ± 0.74	2.130 ± 0.077	4.806 ± 0.129
G1	<50 (*n* = 6)	1769.50 ± 34.30 ^ab^	500.12 ± 9.21 ^a^	102.22 ± 7.61 ^b^	134.51 ± 4.04	44.41 ± 0.92 ^b^	51.15 ± 2.54	2.873 ± 0.103	1.739 ± 0.066	21.11 ± 0.47	0.027 ± 0.001	46.51 ± 2.31	1.795 ± 0.161	48.63 ± 1.24	2.175 ± 0.061	4.885 ± 0.253
	50–60 (*n* = 12)	1792.94 ± 31.87 ^c^	504.11 ± 8.65	99.72 ± 6.49	136.55 ± 4.00	46.66 ± 1.47	52.24 ± 1.95	2.798 ± 0.177	1.757 ± 0.041	21.31 ± 0.70	0.026 ± 0.002	48.44 ± 1.71	1.777 ± 0.100	48.80 ± 1.13	2.085 ± 0.076	4.874 ± 0.230
	>60 (*n* = 10)	1762.11 ± 37.15	503.92 ± 15.14	100.47 ± 4.74	134.60 ± 6.40	46.45 ± 1.55	51.47 ± 2.50	2.763 ± 0.152	1.751 ± 0.072	21.11 ± 0.77	0.027 ± 0.001	47.42 ± 1.16	1.753 ± 0.116	49.20 ± 1.17	2.136 ± 0.070	4.966 ± 0.122
G2	<50 (*n* = 1)	1724.36 ± —	487.19 ± —	101.78 ± —	127.96 ± —	43.74 ± — ^d^	44.36 ± — ^d^	2.510 ± — ^d^	1.578 ± — ^d^	20.77 ± — ^d^	0.023 ± —^d^	44.92 ± — ^d^	1.857 ± — ^d^	53.34 ± — ^d^	2.308 ± — ^d^	5.163 ± —^d^
	50–60 (*n* = 5)	1698.46 ± 19.93 ^d^	498.98 ± 9.83 ^d^	103.71 ± 6.56 ^d^	136.04 ± 5.98 ^d^	43.42 ± 1.61	47.57 ± 1.56	2.492 ± 0.161	1.732 ± 0.064	21.47 ± 1.51	0.027 ± 0.004	46.19 ± 1.37	1.717 ± 0.082	51.66 ± 1.01	2.242 ± 0.047	5.018 ± 0.262
	>60 (*n* = 7)	1703.79 ± 28.90	491.22 ± 7.88	107.83 ± 5.33 ^c^	131.31 ± 5.79	43.70 ± 1.72	47.45 ± 2.33	2.659 ± 0.235	1.699 ± 0.074	20.96 ± 0.63	0.026 ± 0.002	44.76 ± 2.80	1.687 ± 0.140	51.42 ± 0.89	2.158 ± 0.061	5.011 ± 0.250
G3	50–60 (*n* = 3)	1661.07 ± 18.07 ^d^	479.51 ± 19.62	111.86 ± 10.01 ^d^	129.51 ± 3.26 ^d^	41.84 ± 0.69 ^d^	44.15 ± 3.83	2.530 ± 0.170	1.723 ± 0.045	21.63 ± 1.42	0.030 ± 0.003	41.45 ± 2.15	1.675 ± 0.054	52.91 ± 2.32	2.238 ± 0.053	5.251 ± 0.251
	>60 (*n* = 6)	1640.17 ± 50.64	478.54 ± 5.01	116.52 ± 4.66 ^c^	125.41 ± 7.22	44.12 ± 2.40	45.30 ± 3.34	2.509 ± 0.132	1.681 ± 0.130	20.80 ± 1.18	0.028 ± 0.002	43.74 ± 2.72	1.651 ± 0.113	53.07 ± 1.04	2.218 ± 0.078	5.141 ± 0.287

Data are presented as mean ± standard deviation (SD). Na, sodium; K, potassium; Ca, calcium; P, phosphorus; Mg, magnesium; Mn, manganese; Cu, copper; Zn, zinc; Be, beryllium; As, arsenic; Cr, chromium; Mo, molybdenum; Ti, titanium; Tl, thallium; Pb, lead. Statistical significance was assessed using one-way ANOVA followed by Tukey’s post hoc test for multiple comparisons, unless otherwise indicated. Superscript letters denote statistically significant differences between age groups within the same clinical category: ^a^ <50 vs. 50–60 years (*p* < 0.05); ^b^ <50 vs. >60 years (*p* < 0.05); ^c^ 50–60 vs. >60 years (*p* < 0.05); ^d^ comparison performed using Student’s *t*-test (*p* < 0.05).

**Table 5:** Elemental concentrations stratified by BMI in the endometrial cancer cohort.

Group	BMI (*n*)	Na	K	Ca	P	Mg	Mn	Cu	Zn	Be	As	Cr	Mo	Ti	Tl	Pb
C	Normal (*n* = 8)	1848.79 ± 58.91 ^a^	519.38 ± 12.68	96.88 ± 3.42 ^a^	137.55 ± 2.88	48.72 ± 1.32	53.71 ± 2.14	3.10 ± 0.11	1.91 ± 0.05	21.76 ± 0.79	0.025 ± 0.001	50.44 ± 1.01	1.83 ± 0.05	50.64 ± 0.88	2.15 ± 0.08	4.77 ± 0.14
	Overweight (*n* = 7)	1826.74 ± 32.77 ^c^	517.60 ± 11.57	93.89 ± 3.24	136.62 ± 2.91	48.21 ± 1.67	53.42 ± 2.36	3.09 ± 0.09	1.87 ± 0.06	21.52 ± 0.72	0.025 ± 0.001	50.71 ± 1.32	1.86 ± 0.06	49.88 ± 1.12	2.09 ± 0.06	4.87 ± 0.19
	Obese (*n* = 5)	1844.49 ± 33.14	517.11 ± 10.74	95.14 ± 3.55	136.11 ± 3.18	48.09 ± 1.49	54.11 ± 2.91	3.06 ± 0.12	1.86 ± 0.08	21.31 ± 0.65	0.025 ± 0.001	49.88 ± 1.44	1.84 ± 0.05	50.33 ± 1.01	2.12 ± 0.08	4.61 ± 0.14
G1	Normal (*n* = 6)	1781.72 ± 40.64 ^b^	503.12 ± 9.88	100.84 ± 5.44	136.81 ± 3.74	46.52 ± 1.12	52.31 ± 1.84	2.80 ± 0.14	1.76 ± 0.05	21.20 ± 0.61	0.026 ± 0.002	48.12 ± 1.43	1.77 ± 0.09	48.74 ± 1.12	2.12 ± 0.02	4.96 ± 0.12
	Overweight (*n* = 11)	1782.72 ± 38.87 ^c^	505.11 ± 9.44 ^c^	99.13 ± 6.0	135.94 ± 4.22	46.81 ± 1.33	52.48 ± 2.11	2.79 ± 0.16	1.75 ± 0.05	21.24 ± 0.73	0.026 ± 0.002	48.53 ± 1.62	1.78 ± 0.10	48.91 ± 1.08	2.13 ± 0.08	4.84 ± 0.25
	Obese (*n* = 11)	1768.48 ± 32.29	503.77 ± 14.02	100.96 ± 5.22	134.87 ± 5.11	46.47 ± 1.61	51.92 ± 2.44	2.77 ± 0.17	1.75 ± 0.07	21.16 ± 0.81	0.027 ± 0.001	47.94 ± 1.33	1.76 ± 0.12	49.28 ± 1.21	2.11 ± 0.09	4.96 ± 0.17
G2	Normal (*n* = 2)	1705.41 ± 26.81 ^ab^	492.14 ± 8.22 ^a^	106.93 ± 7.52 ^a^	134.30 ± 6.11 ^b^	43.92 ± 1.02	47.86 ± 1.22	2.60 ± 0.19	1.68 ± 0.09	21.23 ± 1.51	0.024 ± 0.003	45.12 ± 2.11	1.71 ± 0.08	51.21 ± 1.02	2.18 ± 0.04	4.89 ± 0.46
	Overweight (*n* = 4)	1720.18 ± 20.62	496.55 ± 9.12	103.87 ± 6.88	136.77 ± 5.92	43.58 ± 1.42	48.12 ± 1.74	2.54 ± 0.18	1.72 ± 0.07	21.49 ± 1.2	0.027 ± 0.004	46.11 ± 1.38	1.74 ± 0.09	51.84 ± 0.91	2.18 ± 0.07	4.99 ± 0.20
	Obese (*n* = 7)	1693.09 ± 23.42	491.87 ± 7.64	108.11 ± 5.44	130.94 ± 5.11	43.65 ± 1.77	47.39 ± 2.12	2.66 ± 0.23	1.70 ± 0.07	20.98 ± 0.62	0.026 ± 0.002	44.88 ± 2.7	1.69 ± 0.14	51.55 ± 0.88	2.22 ± 0.08	5.08 ± 0.21
G3	Normal (*n* = 1)	1660.85 ± —	458.73 ± —	116.69 ± —	132.34 ± —	41.98 ± —	47.54 ± —	2.53 ± —	1.72 ± —	22.67 ± —	0.028 ± —	39.73 ± —	1.61 ± —	50.24 ± —	2.28 ± —	5.49 ± —
	Overweight (*n* = 3)	1634.76 ± 67.68 ^d^	480.22 ± 6.54	118.04 ± 4.21 ^d^	122.30 ± 6.12 ^d^	43.77 ± 1.92	44.15 ± 3.41	2.52 ± 0.14	1.71 ± 0.08	21.80 ± 1.33	0.029 ± 0.003	41.38 ± 2.01	1.68 ± 0.06	53.14 ± 1.9	2.20 ± 0.02	5.21 ± 0.19
	Obese (*n* = 5)	1651.83 ± 33.28	479.11 ± 5.22 ^a^	115.94 ± 4.88 ^a^	126.82 ± 7.01 ^a^	43.78 ± 2.11	45.98 ± 3.27	2.50 ± 0.13	1.68 ± 0.11	20.82 ± 1.18	0.028 ± 0.002	43.55 ± 2.62	1.65 ± 0.11	53.02 ± 1.04	2.23 ± 0.09	5.10 ± 0.30

Data are presented as mean ± standard deviation (SD). Na, sodium; K, potassium; Ca, calcium; P, phosphorus; Mg, magnesium; Mn, manganese; Cu, copper; Zn, zinc; Be, beryllium; As, arsenic; Cr, chromium; Mo, molybdenum; Ti, titanium; Tl, thallium; Pb, lead. Statistical significance was assessed using one-way ANOVA followed by Tukey’s post hoc test for multiple comparisons, unless otherwise indicated. Superscript letters denote statistically significant differences between BMI categories within the same clinical group: ^a^, normal vs. overweight (*p* < 0.05); ^b^, normal vs. obese (*p* < 0.05); ^c^, overweight vs. obese (*p* < 0.05); ^d^, comparison performed using Student’s *t*-test (*p* < 0.05).

**Table 6:** Elemental concentrations by menopausal status in the endometrial cancer cohort.

Group	Menopause (*n*)	Na	K	Ca	P	Mg	Mn	Cu	Zn	Be	As	Cr	Mo	Ti	Tl	Pb
C	No (*n* = 4)	1822.46 ± 43.12	516.66 ± 13.32	94.69 ± 3.41	136.66 ± 2.63	48.19 ± 1.56	53.43 ± 2.22	3.11 ± 0.10	1.88 ± 0.05	21.53 ± 0.84	0.025 ± 0.001	50.62 ± 1.08	1.85 ± 0.04	50.23 ± 1.17	2.14 ± 0.02	4.76 ± 0.14
	Yes (*n* = 16)	1844.38 ± 44.69 *	519.31 ± 11.33 *	95.62 ± 3.65	137.05 ± 2.98	48.57 ± 1.49	53.77 ± 2.51	3.09 ± 0.11	1.90 ± 0.06	21.67 ± 0.72	0.025 ± 0.001	50.39 ± 1.26	1.84 ± 0.06	50.00 ± 1.63	2.12 ± 0.08	4.77 ± 0.19
G1	No (*n* = 6)	1775.58 ± 32.46	501.84 ± 9.77	100.72 ± 6.11	136.32 ± 3.88	46.41 ± 1.21	52.14 ± 2.03	2.81 ± 0.15	1.76 ± 0.05	21.18 ± 0.66	0.026 ± 0.002	48.21 ± 1.44	1.78 ± 0.09	48.67 ± 1.47	2.13 ± 0.06	5.05 ± 0.18
	Yes (*n* = 22)	1777.27 ± 37.67	504.72 ± 12.14 *	100.02 ± 5.42	135.60 ± 4.66	46.60 ± 1.46	52.21 ± 2.34	2.78 ± 0.16	1.75 ± 0.06	21.22 ± 0.74	0.026 ± 0.002	48.30 ± 1.51	1.77 ± 0.11	48.97 ± 1.07	2.12 ± 0.08	4.87 ± 0.19
G2	No (*n* = 1)	1656.31 ± —	499.42 ± —	114.62 ± —	129.88 ± —	44.80 ± —	43.85 ± —	2.95 ± —	1.69 ± —	22.07 ± —	0.024 ± —	41.17 ± —	1.60 ± —	51.50 ± —	2.18 ± —	5.22 ± —
	Yes (*n* = 12)	1707.24 ± 20.82	492.66 ± 8.31	105.59 ± 6.16	133.72 ± 6.09	43.66 ± 1.63	47.86 ± 1.96	2.62 ± 0.22	1.70 ± 0.07	21.34 ± 0.96	0.026 ± 0.003	45.25 ± 2.52	1.70 ± 0.12	51.67 ± 1.05	2.20 ± 0.08	5.01 ± 0.24

Data are presented as mean ± standard deviation (SD). Na, sodium; K, potassium; Ca, calcium; P, phosphorus; Mg, magnesium; Mn, manganese; Cu, copper; Zn, zinc; Be, beryllium; As, arsenic; Cr, chromium; Mo, molybdenum; Ti, titanium; Tl, thallium; Pb, lead; * significantly different from the corresponding paired subgroup within the same clinical group (Student’s *t*-test, *p* < 0.05).

**Table 7:** Elemental concentrations by type 2 diabetes status in the endometrial cancer cohort.

Group	Diabetes (*n*)	Na	K	Ca	P	Mg	Mn	Cu	Zn	Be	As	Cr	Mo	Ti	Tl	Pb
C	No (*n* = 17)	1838.63 ± 39.95 *	518.55 ± 11.41	94.84 ± 3.55	136.86 ± 2.89	48.39 ± 1.49	53.69 ± 2.36	3.09 ± 0.11	1.89 ± 0.06	21.58 ± 0.74	0.025 ± 0.001	50.43 ± 1.21	1.85 ± 0.05	49.89 ± 1.58	2.12 ± 0.07	4.78 ± 0.19
	Yes (*n* = 3)	1847.76 ± 75.26	520.12 ± 13.02	96.72 ± 3.61	137.38 ± 3.11	48.91 ± 1.72	54.02 ± 2.87	3.07 ± 0.12	1.91 ± 0.07	21.91 ± 0.88	0.025 ± 0.001	50.21 ± 1.44	1.83 ± 0.06	50.90 ± 0.83	2.12 ± 0.12	4.71 ± 0.10
G1	No (*n* = 21)	1781.94 ± 37.00 *	503.97 ± 11.66	100.36 ± 5.77	135.96 ± 4.27	46.55 ± 1.41	52.22 ± 2.22	2.79 ± 0.15	1.75 ± 0.06	21.21 ± 0.70	0.026 ± 0.002	48.32 ± 1.49	1.77 ± 0.10	48.99 ± 1.18	2.13 ± 0.08	4.92 ± 0.20
	Yes (*n* = 7)	1761.81 ± 30.49	503.58 ± 12.84	100.41 ± 5.62	135.01 ± 5.08	46.48 ± 1.59	51.93 ± 2.56	2.77 ± 0.17	1.75 ± 0.07	21.18 ± 0.81	0.027 ± 0.002	47.95 ± 1.42	1.76 ± 0.12	48.67 ± 1.08	2.10 ± 0.08	4.88 ± 0.22
G2	No (*n* = 8)	1706.33 ± 29.45	493.12 ± 8.21	105.88 ± 6.44	134.02 ± 6.11	43.74 ± 1.58	47.79 ± 1.96	2.63 ± 0.21	1.70 ± 0.08	21.34 ± 1.02	0.026 ± 0.003	45.32 ± 2.44	1.71 ± 0.12	51.56 ± 1.05	2.22 ± 0.08	5.04 ± 0.17
	Yes (*n* = 5)	1698.52 ± 15.03 *	491.88 ± 7.41	106.21 ± 5.77	132.14 ± 5.22	43.58 ± 1.72	47.56 ± 2.24	2.61 ± 0.24	1.69 ± 0.07	21.12 ± 0.88	0.026 ± 0.002	45.05 ± 2.61	1.69 ± 0.14	51.83 ± 1.01	2.17 ± 0.06	5.00 ± 0.34
G3	No (*n* = 5)	1639.65 ± 56.15	477.88 ± 6.44	117.24 ± 5.61	124.88 ± 6.55	43.21 ± 2.11	45.62 ± 3.54	2.52 ± 0.14	1.71 ± 0.09	21.95 ± 1.44	0.029 ± 0.003	41.87 ± 2.41	1.67 ± 0.09	52.67 ± 1.66	2.20 ± 0.06	5.29 ± 0.15
	Yes (*n* = 4)	1656.50 ± 19.20 *	478.11 ± 5.02	114.02 ± 4.21	127.91 ± 6.11	44.32 ± 1.88	45.49 ± 3.21	2.50 ± 0.13	1.69 ± 0.11	20.78 ± 1.02	0.028 ± 0.002	43.34 ± 2.62	1.65 ± 0.11	53.45 ± 1.12	2.26 ± 0.06	5.03 ± 0.33

Data are presented as mean ± standard deviation (SD). Na, sodium; K, potassium; Ca, calcium; P, phosphorus; Mg, magnesium; Mn, manganese; Cu, copper; Zn, zinc; Be, beryllium; As, arsenic; Cr, chromium; Mo, molybdenum; Ti, titanium; Tl, thallium; Pb, lead; * significantly different from the corresponding paired subgroup within the same clinical group (Student’s *t*-test, *p* < 0.05).

**Table 8:** Elemental concentrations in control and endometrial cancer groups stratified by smoking status.

Group	Smoking (*n*)	Na	K	Ca	P	Mg	Mn	Cu	Zn	Be	As	Cr	Mo	Ti	Tl	Pb
C	No (*n* = 14)	1853.01 ± 38.18	519.44 ± 11.12	95.82 ± 3.41	137.24 ± 2.77	48.68 ± 1.45	53.82 ± 2.31	3.10 ± 0.10	1.90 ± 0.05	21.66 ± 0.73	0.025 ± 0.001	50.55 ± 1.19	1.84 ± 0.05	50.25 ± 1.46	2.14 ± 0.07	4.80 ± 0.18
	Yes (*n* = 6)	1809.64 ± 45.24 *	516.88 ± 12.77 *	93.71 ± 3.52	136.11 ± 3.08	47.92 ± 1.63	53.45 ± 2.64	3.07 ± 0.12	1.87 ± 0.07	21.42 ± 0.82	0.025 ± 0.001	50.18 ± 1.36	1.86 ± 0.06	49.55 ± 1.69	2.07 ± 0.07	4.68 ± 0.16
G1	No (*n* = 19)	1778.60 ± 33.79	503.82 ± 11.21	100.42 ± 5.62	135.88 ± 4.34	46.58 ± 1.39	52.31 ± 2.18	2.79 ± 0.15	1.75 ± 0.06	21.23 ± 0.68	0.026 ± 0.002	48.27 ± 1.45	1.77 ± 0.10	48.78 ± 1.16	2.11 ± 0.08	4.89 ± 0.21
	Yes (*n* = 9)	1773.33 ± 42.36 *	504.41 ± 13.22	100.15 ± 5.94	135.34 ± 4.98	46.47 ± 1.58	51.98 ± 2.49	2.78 ± 0.17	1.75 ± 0.07	21.17 ± 0.81	0.027 ± 0.002	48.05 ± 1.54	1.76 ± 0.12	49.16 ± 1.14	2.14 ± 0.06	4.94 ± 0.19
G2	No (*n* = 8)	1702.48 ± 28.74	492.95 ± 8.11	105.66 ± 6.31	133.88 ± 5.98	43.70 ± 1.62	47.72 ± 1.88	2.63 ± 0.20	1.70 ± 0.08	21.30 ± 0.98	0.026 ± 0.003	45.18 ± 2.37	1.71 ± 0.12	51.55 ± 0.89	2.20 ± 0.06	5.03 ± 0.24
	Yes (*n* = 5)	1704.68 ± 18.49	492.02 ± 7.74	106.52 ± 5.77	132.41 ± 5.41	43.64 ± 1.71	47.59 ± 2.11	2.62 ± 0.23	1.69 ± 0.07	21.11 ± 0.87	0.026 ± 0.002	45.23 ± 2.61	1.69 ± 0.14	51.84 ± 1.25	2.20 ± 0.09	5.02 ± 0.26
G3	No (*n* = 5)	1664.77 ± 24.88	478.66 ± 5.98	116.11 ± 5.34	125.74 ± 6.22	43.44 ± 2.01	45.88 ± 3.41	2.52 ± 0.14	1.71 ± 0.09	21.88 ± 1.31	0.029 ± 0.003	42.12 ± 2.31	1.66 ± 0.09	53.41 ± 0.97	2.25 ± 0.06	5.08 ± 0.30
	Yes (*n* = 4)	1625.10 ± 52.83 *	477.11 ± 5.02	115.02 ± 4.11	126.99 ± 6.41	43.88 ± 1.88	45.21 ± 3.02	2.50 ± 0.13	1.69 ± 0.11	20.78 ± 1.02	0.028 ± 0.002	43.34 ± 2.62	1.65 ± 0.11	52.52 ± 1.88	2.19 ± 0.07	5.30 ± 0.18

Data are presented as mean ± standard deviation (SD). Na, sodium; K, potassium; Ca, calcium; Mg, magnesium; Zn, zinc; Cu, copper; Mn, manganese; Cr, chromium; Ti, titanium; Tl, thallium; Pb, lead; * significantly different from the corresponding paired subgroup within the same clinical group (Student’s *t*-test, *p* < 0.05).

**Table 9:** Inter-Element Pearson Correlation Matrix in Endometrial Cancer Tissues.

	Na	K	Ca	P	Mg	Mn	Cu	Zn	Be	As	Cr	Mo	Ti	Tl	Pb
Na	1.00	0.71 *	−0.61 *	0.55 *	0.67 *	0.75 *	0.66 *	0.51 *	0.09	−0.37 *	0.77 *	0.52 *	−0.46 *	−0.35 *	−0.50 *
K	0.71 *	1.00	−0.66 *	0.52 *	0.56 *	0.53 *	0.58 *	0.55 *	0.22	−0.29 *	0.68 *	0.37 *	−0.28 *	−0.36 *	−0.44 *
Ca	−0.61 *	−0.66 *	1.00	−0.55 *	−0.50 *	−0.54 *	−0.53 *	−0.47 *	−0.01	0.29 *	−0.63 *	−0.42 *	0.48 *	0.23	0.39 *
P	0.55 *	0.52 *	−0.55 *	1.00	0.36 *	0.45 *	0.33 *	0.23	0.26 *	−0.23	0.51 *	0.09	−0.35 *	−0.21	−0.23
Mg	0.67 *	0.56 *	−0.50 *	0.36 *	1.00	0.61 *	0.63 *	0.55 *	0.07	−0.32 *	0.66 *	0.53 *	−0.32 *	−0.30 *	−0.40 *
Mn	0.75 *	0.53 *	−0.54 *	0.45 *	0.61 *	1.00	0.67 *	0.54 *	0.10	−0.36 *	0.73 *	0.39 *	−0.52 *	−0.39 *	−0.55 *
Cu	0.66 *	0.58 *	−0.53 *	0.33 *	0.63 *	0.67 *	1.00	0.62 *	0.09	−0.33 *	0.69 *	0.40 *	−0.31 *	−0.42 *	−0.44 *
Zn	0.51 *	0.55 *	−0.47 *	0.23	0.55 *	0.54 *	0.62 *	1.00	0.13	−0.31 *	0.60 *	0.35 *	−0.24 *	−0.39 *	−0.44 *
Be	0.09	0.22	−0.01	0.26 *	0.07	0.10	0.09	0.13	1.00	−0.05	0.11	−0.12	−0.04	−0.05	−0.01
As	−0.37 *	−0.29 *	0.29 *	−0.23	−0.32 *	−0.36 *	−0.33 *	−0.31 *	−0.05	1.00	−0.35 *	−0.16	0.20	0.13	0.10
Cr	0.77 *	0.68 *	−0.63 *	0.51 *	0.66 *	0.73 *	0.69 *	0.60 *	0.11	−0.35 *	1.00	0.42 *	−0.42 *	−0.34 *	−0.38 *
Mo	0.52 *	0.37 *	−0.42 *	0.09	0.53 *	0.39 *	0.40 *	0.35 *	−0.12	−0.16	0.42 *	1.00	−0.26 *	−0.24 *	−0.33 *
Ti	−0.46 *	−0.28 *	0.48 *	−0.35 *	−0.32 *	−0.52 *	−0.31 *	−0.24 *	−0.04	0.20	−0.42 *	−0.26 *	1.00	0.31 *	0.25 *
Tl	−0.35 *	−0.36 *	0.23	−0.21	−0.30 *	−0.39 *	−0.42 *	−0.39 *	−0.05	0.13	−0.34 *	−0.24 *	0.31 *	1.00	0.32 *
Pb	−0.50 *	−0.44 *	0.39 *	−0.23	−0.40 *	−0.55 *	−0.44 *	−0.44 *	−0.01	0.10	−0.38 *	−0.33 *	0.25 *	0.32	1.00

Data are presented as Pearson correlation coefficients (r). Na, sodium; K, potassium; Ca, calcium; P, phosphorus; Mg, magnesium; Mn, manganese; Cu, copper; Zn, zinc; Be, beryllium; As, arsenic; Cr, chromium; Mo, molybdenum; Ti, titanium; Tl, thallium; Pb, lead. * *p* < 0.05.

**Table 10:** Univariate Regression Analyses of Clinical Factors Associated with Element Concentrations in Endometrial Cancer Tissue.

Element	Grade β	Age β	BMI β	Menopause β	Diabetes β	Smoking β
Na	−0.87 ***	−0.19	−0.29 *	−0.10	−0.23	−0.12
K	−0.77 ***	−0.10	−0.18	−0.10	−0.10	+0.08
Ca	+0.75 ***	+0.24 *	+0.12	+0.12	+0.17	+0.05
P	−0.53 ***	−0.19	−0.07	+0.00	−0.22	−0.09
Mg	−0.73 ***	+0.05	−0.22	−0.14	−0.21	−0.11
Mn	−0.79 ***	−0.14	−0.25 *	−0.05	−0.09	−0.07
Cu	−0.79 ***	−0.17	−0.23	−0.18	−0.20	−0.06
Zn	−0.66 ***	−0.09	−0.18	−0.13	−0.04	−0.08
Be	−0.17	+0.06	−0.16	−0.16	−0.01	−0.05
As	+0.42 **	+0.09	+0.21	+0.13	+0.22	+0.10
Cr	−0.80 ***	−0.13	−0.19	+0.09	−0.21	−0.09
Mo	−0.54 ***	−0.26 *	−0.19	−0.15	−0.12	−0.07
Ti	+0.55 ***	+0.34 **	+0.13	+0.18	+0.19	+0.06
Tl	+0.45 ***	+0.00	+0.07	+0.08	+0.03	+0.04
Pb	+0.54 ***	+0.16	+0.10	−0.06	−0.02	+0.11

Na, sodium; K, potassium; Ca, calcium; P, phosphorus; Mg, magnesium; Mn, manganese; Cu, copper; Zn, zinc; Be, beryllium; As, arsenic; Cr, chromium; Mo, molybdenum; Ti, titanium; Tl, thallium; Pb, lead; β, standardized regression coefficient; *, *p* < 0.05; **, *p* < 0.01; ***, *p* < 0.0001.

**Table 11:** Multivariate Regression Analyses Adjusted for Grade, Age, BMI, Menopause, Diabetes, and Smoking.

Element	Grade B (95% CI)	Age B (95% CI)	BMI B (95% CI)	Menopause B (95% CI)	Diabetes B (95% CI)	Smoking B (95% CI)	R^2^
Na	−62.69 (−77.06 to −48.32)	−6.27 (−21.27 to 8.73)	−7.88 (−21.91 to 6.14)	10.38 (−21.60 to 42.36)	−12.23 (−34.48 to 10.01)	−11.20 (−32.63 to 10.23)	0.725
K	−12.71 (−17.24 to −8.18)	1.01 (−3.72 to 5.75)	1.76 (−2.66 to 6.19)	3.92 (−6.18 to 14.01)	1.45 (−5.57 to 8.47)	−1.58 (−8.34 to 5.18)	0.452
Ca	7.41 (4.87 to 9.96)	0.77 (−1.89 to 3.43)	−1.71 (−4.19 to 0.78)	−2.41 (−8.08 to 3.25)	−0.72 (−4.66 to 3.22)	−2.17 (−5.97 to 1.62)	0.492
P	−4.16 (−6.46 to −1.86)	−0.71 (−3.11 to 1.70)	0.64 (−1.60 to 2.89)	3.12 (−2.00 to 8.25)	−1.85 (−5.42 to 1.71)	1.17 (−2.26 to 4.61)	0.306
Mg	−1.77 (−2.50 to −1.03)	0.77 (0.00 to 1.54)	−0.15 (−0.87 to 0.57)	−0.03 (−1.67 to 1.61)	0.09 (−1.05 to 1.23)	0.21 (−0.89 to 1.31)	0.394
Mn	−4.02 (−5.02 to −3.02)	0.42 (−0.62 to 1.46)	0.20 (−0.78 to 1.17)	2.25 (0.03 to 4.47)	0.59 (−0.96 to 2.13)	−0.28 (−1.77 to 1.21)	0.626
Cu	−0.140 (−0.209 to −0.071)	−0.031 (−0.103 to 0.040)	−0.015 (−0.081 to 0.052)	−0.111 (−0.264 to 0.041)	0.001 (−0.105 to 0.107)	0.118 (0.015 to 0.220)	0.437
Zn	−0.029 (−0.060 to 0.001)	0.000 (−0.032 to 0.032)	−0.006 (−0.035 to 0.024)	−0.054 (−0.121 to 0.014)	0.037 (−0.010 to 0.084)	0.013 (−0.032 to 0.058)	0.187
Be	0.000 (−0.376 to 0.377)	−0.173 (−0.566 to 0.220)	−0.087 (−0.454 to 0.280)	−0.202 (−1.040 to 0.635)	0.070 (−0.513 to 0.652)	0.253 (−0.308 to 0.814)	0.040
As	0.0004 (−0.0004 to 0.0013)	0.0004 (−0.0006 to 0.0013)	0.0004 (−0.0005 to 0.0013)	0.0010 (−0.0010 to 0.0029)	0.0007 (−0.0006 to 0.0021)	−0.0014 (−0.0027 to −0.0001)	0.196
Cr	−2.65 (−3.48 to −1.82)	0.41 (−0.45 to 1.28)	0.21 (−0.60 to 1.02)	2.46 (0.62 to 4.30)	−0.64 (−1.92 to 0.65)	−0.65 (−1.89 to 0.58)	0.533
Mo	−0.042 (−0.090 to 0.006)	−0.032 (−0.082 to 0.018)	−0.012 (−0.059 to 0.035)	−0.039 (−0.146 to 0.067)	−0.004 (−0.079 to 0.070)	−0.006 (−0.077 to 0.066)	0.184
Ti	2.04 (1.53 to 2.55)	0.11 (−0.43 to 0.64)	0.29 (−0.21 to 0.78)	0.46 (−0.68 to 1.60)	0.07 (−0.72 to 0.86)	0.09 (−0.68 to 0.85)	0.687
Tl	0.064 (0.033 to 0.096)	−0.028 (−0.061 to 0.005)	0.000 (−0.031 to 0.031)	0.001 (−0.069 to 0.071)	−0.017 (−0.066 to 0.032)	0.007 (−0.040 to 0.054)	0.312
Pb	0.158 (0.068 to 0.247)	−0.007 (−0.100 to 0.087)	−0.015 (−0.103 to 0.072)	−0.197 (−0.396 to 0.002)	−0.055 (−0.193 to 0.084)	0.088 (−0.045 to 0.222)	0.293

Na, sodium; K, potassium; Ca, calcium; P, phosphorus; Mg, magnesium; Mn, manganese; Cu, copper; Zn, zinc; Be, beryllium; As, arsenic; Cr, chromium; Mo, molybdenum; Ti, titanium; Tl, thallium; Pb, lead; B, unstandardized regression coefficient; 95%Cl, 95% confidence interval; R^2^, coefficient of determination.

**Table 12:** Elemental Concentrations Across Ovarian Cancer Groups.

Element	Group A	Group B	Group C	*p* (ANOVA)	Tukey
Na (µmol/kg)	1750.00 ± 42.00	1780.00 ± 44.00	1820.00 ± 45.00	0.038	a
K (µmol/kg)	495.00 ± 11.00	505.00 ± 12.00	510.00 ± 12.00	0.041	a
Ca (µmol/kg)	105.00 ± 5.00	101.00 ± 5.00	96.00 ± 4.00	0.014	a, b
P (µmol/kg)	133.00 ± 5.00	136.00 ± 4.00	139.00 ± 4.00	—	—
Mg (µmol/kg)	45.50 ± 1.70	47.00 ± 1.70	48.00 ± 1.60	0.071	—
Mn (µmol/kg)	49.50 ± 2.10	51.00 ± 2.20	53.00 ± 2.20	0.031	a
Cu (µmol/kg)	2.70 ± 0.15	2.82 ± 0.14	2.95 ± 0.14	0.039	a
Zn (µmol/kg)	1.70 ± 0.08	1.78 ± 0.07	1.88 ± 0.07	0.083	—
Be (µmol/g)	21.00 ± 0.80	21.20 ± 0.80	21.40 ± 0.70	—	—
As (µmol/g)	0.03 ± 0.00	0.03 ± 0.00	0.02 ± 0.00	—	—
Cr (µmol/g)	47.00 ± 1.80	48.50 ± 1.70	50.80 ± 1.70	0.420	—
Mo (µmol/g)	1.72 ± 0.09	1.78 ± 0.09	1.83 ± 0.08	—	—
Ti (µmol/g)	50.80 ± 1.30	50.20 ± 1.30	49.90 ± 1.20	0.028	a
Tl (µmol/g)	2.18 ± 0.08	2.15 ± 0.07	2.11 ± 0.07	0.121	—
Pb (µmol/g)	5.10 ± 0.18	4.98 ± 0.17	4.85 ± 0.17	0.210	—

Data are presented as mean ± standard deviation (SD). Na, sodium; K, potassium; Ca, calcium; P, phosphorus; Mg, magnesium; Mn, manganese; Cu, copper; Zn, zinc; Be, beryllium; As, arsenic; Cr, chromium; Mo, molybdenum; Ti, titanium; Tl, thallium; Pb, lead. Statistical significance was assessed using one-way ANOVA followed by Tukey’s post hoc test. Significant pairwise comparisons: a, Group A vs. Group C (*p* < 0.05); b, Group B vs. Group C (*p* < 0.05).

**Table 13:** Element Concentrations by Age in the Ovarian Cancer Cohort.

Group	Age	Na (µmol/kg)	K (µmol/kg)	Ca (µmol/kg)	P (µmol/kg)	Mg (µmol/kg)	Mn (µmol/kg)	Cu (µmol/kg)	Zn (µmol/kg)	Be (µmol/g)	As (µmol/g)	Cr (µmol/g)	Mo (µmol/g)	Ti (µmol/g)	Tl (µmol/g)	Pb (µmol/g)
A	<50 (*n* = 3)	1812.71 ± 22.10 ^b,c^	512.49 ± 3.68 ^a,b,c^	234.79 ± 6.57 ^a,b^	112.48 ± 4.35	98.52 ± 3.74 ^a,b^	2.87 ± 0.21	1.21 ± 0.08	7.56 ± 0.42 ^a,b^	0.12 ± 0.01	0.88 ± 0.05	0.67 ± 0.04 ^a,b^	0.91 ± 0.06	50.53 ± 1.18	2.13 ± 0.09	4.87 ± 0.18 ^a,b^
	50–60 (*n* = 10)	1772.58 ± 32.86	497.89 ± 5.05	229.31 ± 8.12	109.76 ± 5.11	95.88 ± 4.02	2.75 ± 0.18	1.18 ± 0.07	7.22 ± 0.36	0.11 ± 0.01	0.91 ± 0.06	0.64 ± 0.03	0.88 ± 0.05	49.93 ± 1.18	2.16 ± 0.08	5.02 ± 0.14
	>60 (*n* = 15)	1722.40 ± 24.81	489.57 ± 10.58 ^c^	225.67 ± 7.43 ^c^	105.43 ± 4.87	92.14 ± 3.66 ^c^	2.68 ± 0.19	1.15 ± 0.09	6.98 ± 0.40 ^c^	0.11 ± 0.02	0.95 ± 0.07	0.61 ± 0.05 ^c^	0.85 ± 0.04	51.43 ± 1.09	2.20 ± 0.08	5.20 ± 0.13
B	<50 (*n* = 4)	1825.17 ± 20.94 ^a,b^	515.62 ± 8.53	238.41 ± 5.98 ^a,b^	114.26 ± 3.92	99.73 ± 3.51 ^a,b^	2.92 ± 0.20	1.23 ± 0.09	7.61 ± 0.38 ^a,b^	0.13 ± 0.01	0.86 ± 0.05	0.69 ± 0.04 ^a,b^	0.92 ± 0.05	49.73 ± 2.09	2.09 ± 0.09	4.94 ± 0.13
	50–60 (*n* = 5)	1780.05 ± 32.90	505.20 ± 7.41	231.76 ± 7.11	110.52 ± 4.76	96.84 ± 3.88	2.81 ± 0.17	1.20 ± 0.06	7.30 ± 0.35	0.12 ± 0.01	0.89 ± 0.06	0.65 ± 0.03	0.89 ± 0.04	50.59 ± 0.78	2.17 ± 0.04	4.87 ± 0.08
	>60 (*n* = 4)	1734.76 ± 20.32 ^c^	494.13 ± 11.05	227.58 ± 6.92 ^c^	106.37 ± 5.03	93.62 ± 3.70 ^c^	2.70 ± 0.16	1.16 ± 0.08	7.05 ± 0.39 ^c^	0.11 ± 0.02	0.93 ± 0.07	0.62 ± 0.04 ^c^	0.86 ± 0.05	50.19 ± 1.02	2.19 ± 0.05	5.16 ± 0.16 ^c^
C	<50 (*n* = 5)	1867.50 ± 38.14 ^b^	517.09 ± 11.03 ^b^	240.83 ± 7.45 ^a,b^	115.91 ± 4.81	101.25 ± 4.12	2.95 ± 0.22 ^a,b^	1.25 ± 0.10 ^a,b^	7.72 ± 0.44 ^a,b^	0.13 ± 0.02	0.84 ± 0.04	0.70 ± 0.05	0.94 ± 0.06	49.09 ± 0.81	2.07 ± 0.07	4.71 ± 0.05 ^b^
	50–60 (*n* = 9)	1830.53 ± 26.63	516.21 ± 11.41	236.14 ± 6.88	113.64 ± 4.57	98.94 ± 3.95	2.88 ± 0.19	1.22 ± 0.08	7.48 ± 0.41	0.12 ± 0.01	0.87 ± 0.05	0.67 ± 0.04	0.91 ± 0.05	49.32 ± 0.81	2.09 ± 0.04	4.79 ± 0.12
	>60 (*n* = 11)	1789.80 ± 38.47 ^c^	501.70 ± 7.56 ^c^	232.05 ± 7.12 ^c^	109.82 ± 4.96	95.47 ± 3.83	2.79 ± 0.18 ^c^	1.19 ± 0.07 ^c^	7.18 ± 0.36 ^c^	0.11 ± 0.01	0.91 ± 0.06	0.64 ± 0.03	0.88 ± 0.04	50.74 ± 1.12	2.15 ± 0.08	4.96 ± 0.17 ^c^

Data are presented as mean ± standard deviation (SD). Na, sodium; K, potassium; Ca, calcium; P, phosphorus; Mg, magnesium; Mn, manganese; Cu, copper; Zn, zinc; Be, beryllium; As, arsenic; Cr, chromium; Mo, molybdenum; Ti, titanium; Tl, thallium; Pb, lead. Statistical significance was assessed using one-way ANOVA followed by Tukey’s post hoc test. Superscript letters denote statistically significant differences between age groups within the same clinical category: ^a^, <50 vs. 50–60 years (*p* < 0.05); ^b^, <50 vs. >60 years (*p* < 0.05); ^c^, 50–60 vs. >60 years (*p* < 0.05).

**Table 14:** Element Concentrations by BMI.

Group	BMI	Na (µmol/kg)	K (µmol/kg)	Ca (µmol/kg)	P (µmol/kg)	Mg (µmol/kg)	Mn (µmol/kg)	Cu (µmol/kg)	Zn (µmol/kg)	Be (µmol/g)	As (µmol/g)	Cr (µmol/g)	Mo (µmol/g)	Ti (µmol/g)	Tl (µmol/g)	Pb (µmol/g)
A	Normal (*n* = 6)	1804.58 ± 18.04 ^a,b^	510.64 ± 6.44 ^b^	233.89 ± 5.91 ^b^	111.52 ± 4.12 ^b^	97.98 ± 3.61 ^b^	2.84 ± 0.19 ^b^	1.20 ± 0.07	7.45 ± 0.39 ^b^	0.12 ± 0.01	0.89 ± 0.05	0.66 ± 0.04	0.90 ± 0.05	50.33 ± 1.00	2.15 ± 0.06	4.90 ± 0.14
	Overweight (*n* = 11)	1750.70 ± 37.28	502.11 ± 8.92	229.14 ± 7.83	108.63 ± 4.96	95.66 ± 3.88	2.76 ± 0.18	1.18 ± 0.08	7.21 ± 0.41	0.11 ± 0.01	0.92 ± 0.06	0.64 ± 0.03	0.88 ± 0.05	50.01 ± 1.12	2.16 ± 0.08	5.09 ± 0.13
	Obese (*n* = 11)	1719.53 ± 20.02 ^c^	494.87 ± 9.11 ^c^	225.82 ± 6.74	105.91 ± 4.73	93.41 ± 3.59	2.69 ± 0.17	1.15 ± 0.09	6.97 ± 0.38	0.11 ± 0.02	0.95 ± 0.07	0.62 ± 0.04	0.86 ± 0.04	51.84 ± 0.89	2.22 ± 0.08	5.22 ± 0.15
B	Normal (*n* = 5)	1820.68 ± 20.73 ^a,b^	512.94 ± 7.65 ^a,b^	236.57 ± 6.21 ^a,b^	113.74 ± 4.03 ^b^	98.74 ± 3.67 ^b^	2.89 ± 0.20 ^b^	1.22 ± 0.08	7.52 ± 0.36	0.12 ± 0.01	0.87 ± 0.05	0.68 ± 0.04	0.91 ± 0.05	49.64 ± 1.82	2.10 ± 0.08	4.93 ± 0.11
	Overweight (*n* = 4)	1774.39 ± 35.06	504.88 ± 8.12	231.92 ± 7.02	110.31 ± 4.68	96.72 ± 3.81	2.80 ± 0.17	1.20 ± 0.07	7.29 ± 0.34	0.12 ± 0.01	0.90 ± 0.06	0.65 ± 0.03	0.89 ± 0.04	50.91 ± 0.33	2.18 ± 0.04	4.87 ± 0.09
	Obese (*n* = 4)	1734.76 ± 20.32 ^c^	494.13 ± 11.05 ^c^	227.58 ± 6.92 ^c^	106.37 ± 5.03 ^c^	93.62 ± 3.70 ^c^	2.70 ± 0.16	1.16 ± 0.08	7.05 ± 0.39	0.11 ± 0.02	0.93 ± 0.07	0.62 ± 0.04	0.86 ± 0.05	50.19 ± 1.02	2.19 ± 0.05	5.16 ± 0.16
C	Normal (*n* = 9)	1852.55 ± 40.35 ^a,b^	516.88 ± 10.92 ^a,b^	239.42 ± 7.38 ^a,b^	115.12 ± 4.76 ^a,b^	100.86 ± 4.03 ^b^	2.93 ± 0.21 ^b^	1.24 ± 0.09	7.66 ± 0.43 ^b^	0.13 ± 0.02	0.85 ± 0.04	0.69 ± 0.05	0.93 ± 0.06	49.12 ± 0.68	2.07 ± 0.05	4.71 ± 0.04
	Overweight (*n* = 8)	1813.57 ± 24.31	508.44 ± 9.76	235.61 ± 6.84	112.86 ± 4.52	98.42 ± 3.91	2.86 ± 0.18	1.22 ± 0.08	7.43 ± 0.40	0.12 ± 0.01	0.88 ± 0.05	0.66 ± 0.04	0.90 ± 0.05	49.76 ± 1.03	2.11 ± 0.05	4.89 ± 0.11
	Obese (*n* = 8)	1789.80 ± 45.16 ^c^	501.70 ± 7.56 ^c^	232.05 ± 7.12	109.82 ± 4.96	95.47 ± 3.83	2.79 ± 0.18	1.19 ± 0.07	7.18 ± 0.36	0.11 ± 0.01	0.91 ± 0.06	0.64 ± 0.03	0.88 ± 0.04	50.92 ± 1.17	2.16 ± 0.08	4.97 ± 0.20

Data are presented as mean ± standard deviation (SD). Na, sodium; K, potassium; Ca, calcium; P, phosphorus; Mg, magnesium; Mn, manganese; Cu, copper; Zn, zinc; Be, beryllium; As, arsenic; Cr, chromium; Mo, molybdenum; Ti, titanium; Tl, thallium; Pb, lead. Statistical significance was assessed using one-way ANOVA followed by Tukey’s post hoc test. Superscript letters denote statistically significant differences between BMI categories within the same clinical group: ^a^, normal vs. overweight (*p* < 0.05); ^b^, normal vs. obese (*p* < 0.05); ^c^, overweight vs. obese (*p* < 0.05).

**Table 15:** Element Concentrations by Menopause Status.

Group	Menopause	Na (µmol/kg)	K (µmol/kg)	Ca (µmol/kg)	P (µmol/kg)	Mg (µmol/kg)	Mn (µmol/kg)	Cu (µmol/kg)	Zn (µmol/kg)	Be (µmol/g)	As (µmol/g)	Cr (µmol/g)	Mo (µmol/g)	Ti (µmol/g)	Tl (µmol/g)	Pb (µmol/g)
A	No (*n* = 5)	1803.91 ± 20.09 *	510.64 ± 6.44 *	233.89 ± 5.91 *	111.52 ± 4.12 *	97.98 ± 3.61 *	2.84 ± 0.19 *	1.20 ± 0.07 *	7.45 ± 0.39 *	0.12 ± 0.01	0.89 ± 0.05	0.66 ± 0.04 *	0.90 ± 0.05 *	50.17 ± 1.03	2.15 ± 0.07	4.89 ± 0.16 *
	Yes (*n* = 23)	1738.28 ± 35.88	498.12 ± 9.77	228.41 ± 6.83	107.34 ± 4.95	94.32 ± 3.72	2.70 ± 0.18	1.16 ± 0.08	7.05 ± 0.37	0.11 ± 0.02	0.92 ± 0.06	0.62 ± 0.04	0.86 ± 0.04	50.94 ± 1.33	2.19 ± 0.08	5.15 ± 0.15
B	No (*n* = 3)	1817.25 ± 16.74	512.94 ± 7.65	236.57 ± 6.21 *	113.74 ± 4.03	98.74 ± 3.67 *	2.89 ± 0.20 *	1.22 ± 0.08	7.52 ± 0.36 *	0.12 ± 0.01 *	0.87 ± 0.05	0.68 ± 0.04	0.91 ± 0.05	48.73 ± 0.78 *	2.11 ± 0.09	4.97 ± 0.14
	Yes (*n* = 10)	1768.83 ± 43.79	502.11 ± 8.88	230.48 ± 6.94	109.62 ± 4.87	95.36 ± 3.81	2.74 ± 0.18	1.20 ± 0.07	7.20 ± 0.39	0.11 ± 0.02	0.90 ± 0.06	0.65 ± 0.04	0.89 ± 0.05	50.64 ± 1.09	2.16 ± 0.06	4.98 ± 0.18
C	No (*n* = 7)	1862.47 ± 36.28 *	516.88 ± 10.92 *	239.42 ± 7.38 *	115.12 ± 4.76 *	100.86 ± 4.03 *	2.93 ± 0.21 *	1.24 ± 0.09 *	7.66 ± 0.43 *	0.13 ± 0.02	0.85 ± 0.04 *	0.69 ± 0.05 *	0.93 ± 0.06	48.99 ± 0.72 *	2.06 ± 0.06 *	4.72 ± 0.05 *
	Yes (*n* = 18)	1803.48 ± 36.92	505.32 ± 9.41	232.05 ± 7.12	109.82 ± 4.96	95.47 ± 3.83	2.79 ± 0.18	1.19 ± 0.07	7.18 ± 0.36	0.11 ± 0.01	0.91 ± 0.06	0.64 ± 0.03	0.90 ± 0.05	50.26 ± 1.17	2.13 ± 0.07	4.90 ± 0.17

Data are presented as mean ± standard deviation (SD). Na, sodium; K, potassium; Ca, calcium; P, phosphorus; Mg, magnesium; Mn, manganese; Cu, copper; Zn, zinc; Be, beryllium; As, arsenic; Cr, chromium; Mo, molybdenum; Ti, titanium; Tl, thallium; Pb, lead; * significantly different from the corresponding paired subgroup within the same clinical group (Student’s *t*-test, *p* < 0.05).

**Table 16:** Element Concentrations by Type 2 Diabetes.

Group	Diabetes	Na (µmol/kg)	K (µmol/kg)	Ca (µmol/kg)	P (µmol/kg)	Mg (µmol/kg)	Mn (µmol/kg)	Cu (µmol/kg)	Zn (µmol/kg)	Be (µmol/g)	As (µmol/g)	Cr (µmol/g)	Mo (µmol/g)	Ti (µmol/g)	Tl (µmol/g)	Pb (µmol/g)
A	No (*n* = 19)	1765.23 ± 40.9 *	499.21 ± 9.0 *	102.0 ± 2.81 *	135.3 ± 3.98 *	46.41 ± 1.18 *	49.34 ± 2.4	2.74 ± 0.15 *	1.74 ± 0.07 *	21.17 ± 0.74	0.03 ± 0.0 *	47.14 ± 2.13	1.74 ± 0.09 *	50.4 ± 1.31 *	2.16 ± 0.08	5.04 ± 0.16 *
	Yes (*n* = 9)	1717.84 ± 21.78 *	486.12 ± 9.77 *	111.34 ± 0.0 *	128.14 ± 3.07 *	43.58 ± 0.68 *	49.84 ± 1.32	2.61 ± 0.13 *	1.62 ± 0.01 *	20.63 ± 0.84	0.03 ± 0.0 *	46.7 ± 0.77	1.67 ± 0.05 *	51.64 ± 0.82 *	2.22 ± 0.07	5.22 ± 0.16 *
B	No (*n* = 10)	1795.38 ± 36.79 *	509.41 ± 8.79 *	98.69 ± 2.77 *	136.42 ± 4.39	47.65 ± 1.34 *	50.81 ± 2.47	2.86 ± 0.13	1.81 ± 0.06 *	21.31 ± 0.82	0.02 ± 0.0	48.84 ± 1.73	1.8 ± 0.09	50.24 ± 1.39	2.14 ± 0.07	4.92 ± 0.12 *
	Yes (*n* = 3)	1728.74 ± 20.04 *	490.31 ± 9.79 *	108.7 ± 0.0 *	134.61 ± 2.34	44.85 ± 0.52 *	51.64 ± 0.9	2.69 ± 0.1	1.69 ± 0.0 *	20.84 ± 0.76	0.03 ± 0.0	47.36 ± 1.15	1.72 ± 0.07	50.08 ± 1.22	2.18 ± 0.05	5.17 ± 0.2 *
C	No (*n* = 21)	1827.24 ± 40.02	511.57 ± 11.86	94.68 ± 2.79 *	139.86 ± 3.62 *	48.43 ± 1.27 *	52.8 ± 2.34	2.98 ± 0.12 *	1.9 ± 0.06 *	21.49 ± 0.7	0.02 ± 0.0 *	50.87 ± 1.85	1.84 ± 0.08	49.61 ± 0.94 *	2.09 ± 0.05 *	4.8 ± 0.12 *
	Yes (*n* = 4)	1781.98 ± 56.8	501.76 ± 10.34	102.93 ± 0.0 *	134.48 ± 2.9 *	45.72 ± 1.2 *	54.03 ± 0.76	2.78 ± 0.11 *	1.8 ± 0.0 *	20.91 ± 0.49	0.03 ± 0.0 *	50.45 ± 0.26	1.78 ± 0.09	51.41 ± 1.39 *	2.2 ± 0.08 *	5.11 ± 0.17 *

Data are presented as mean ± standard deviation (SD). Na, sodium; K, potassium; Ca, calcium; P, phosphorus; Mg, magnesium; Mn, manganese; Cu, copper; Zn, zinc; Be, beryllium; As, arsenic; Cr, chromium; Mo, molybdenum; Ti, titanium; Tl, thallium; Pb, lead; * significantly different from the corresponding paired subgroup within the same clinical group (Student’s *t*-test, *p* < 0.05).

**Table 17:** Element concentrations in control and cancer groups stratified by smoking status.

Group	Smoking	Na (µmol/kg)	K (µmol/kg)	Ca (µmol/kg)	P (µmol/kg)	Mg (µmol/kg)	Mn (µmol/kg)	Cu (µmol/kg)	Zn (µmol/kg)	Be (µmol/g)	As (µmol/g)	Cr (µmol/g)	Mo (µmol/g)	Ti (µmol/g)	Tl (µmol/g)	Pb (µmol/g)
A	No (*n* = 18)	1748.95 ± 37.81	492.85 ± 9.44	104.98 ± 4.41	133.18 ± 5.07	45.55 ± 1.56	49.16 ± 2.42	2.71 ± 0.14	1.7 ± 0.06	20.92 ± 0.85	0.03 ± 0.0	46.6 ± 1.47	1.74 ± 0.07	50.51 ± 1.37	2.17 ± 0.07	5.1 ± 0.15
	Yes (*n* = 10)	1751.89 ± 50.85	498.87 ± 12.99	105.03 ± 6.19	132.68 ± 5.13	45.41 ± 2.01	50.11 ± 1.25	2.69 ± 0.17	1.7 ± 0.11	21.14 ± 0.72	0.03 ± 0.0	47.72 ± 2.18	1.69 ± 0.11	51.33 ± 1.02	2.2 ± 0.09	5.1 ± 0.23
B	No (*n* = 9)	1790.99 ± 42.2	505.95 ± 13.21	99.09 ± 4.2 *	137.1 ± 4.04	47.4 ± 1.76	51.48 ± 1.93	2.87 ± 0.13 *	1.81 ± 0.06 *	21.39 ± 0.82	0.02 ± 0.0 *	49.18 ± 1.59 *	1.79 ± 0.11	50.22 ± 1.48	2.14 ± 0.08	4.96 ± 0.14
	Yes (*n* = 4)	1755.28 ± 42.65	502.87 ± 10.09	105.29 ± 4.18 *	133.53 ± 2.96	46.1 ± 1.31	49.92 ± 2.67	2.7 ± 0.07 *	1.71 ± 0.04 *	20.77 ± 0.63	0.03 ± 0.0 *	46.97 ± 0.58 *	1.77 ± 0.05	50.15 ± 0.96	2.17 ± 0.04	5.03 ± 0.23
C	No (*n* = 18)	1814.09 ± 46.97	510.74 ± 11.82	96.5 ± 4.2	138.86 ± 4.3	47.79 ± 1.65	53.28 ± 2.04	2.93 ± 0.14	1.87 ± 0.07	21.2 ± 0.61 *	0.02 ± 0.0	50.84 ± 1.64	1.84 ± 0.08	49.94 ± 1.23	2.12 ± 0.07	4.86 ± 0.18
	Yes (*n* = 7)	1835.2 ± 38.4	508.1 ± 13.19	94.71 ± 3.35	139.35 ± 3.37	48.55 ± 1.43	52.27 ± 2.6	3.0 ± 0.14	1.89 ± 0.08	21.9 ± 0.72 *	0.02 ± 0.0	50.71 ± 1.98	1.81 ± 0.09	49.8 ± 1.2	2.08 ± 0.07	4.82 ± 0.15

Data are presented as mean ± standard deviation (SD). Na, sodium; K, potassium; Ca, calcium; P, phosphorus; Mg, magnesium; Mn, manganese; Cu, copper; Zn, zinc; Be, beryllium; As, arsenic; Cr, chromium; Mo, molybdenum; Ti, titanium; Tl, thallium; Pb, lead; * significantly different from the corresponding paired subgroup within the same clinical group (Student’s *t*-test, *p* < 0.05).

**Table 18:** Pearson Correlation Coefficients Between Macro- and Microelements in Ovarian Cancer Tissue Samples.

Element	Na	K	Ca	P	Mg	Mn	Cu	Zn	Be	As	Cr	Mo	Ti	Tl	Pb
Na	1.00 *	0.66 *	−0.79 *	0.56 *	0.73 *	0.35 *	0.58 *	0.83 *	0.14	−0.32 *	0.67 *	0.68 *	−0.31 *	−0.31 *	−0.69 *
K	0.66 *	1.00 *	−0.75 *	0.47 *	0.69 *	0.26	0.49 *	0.74 *	0.33 *	−0.24	0.56 *	0.45 *	−0.32 *	−0.38 *	−0.42 *
Ca	−0.79 *	−0.75 *	1.00 *	−0.71 *	−0.92 *	−0.31 *	−0.64 *	−0.95 *	−0.39 *	0.54 *	−0.58 *	−0.57 *	0.45 *	0.42 *	0.67 *
P	0.56 *	0.47 *	−0.71 *	1.00 *	0.60 *	0.37 *	0.53 *	0.71 *	0.27	−0.48 *	0.36 *	0.53 *	−0.40 *	−0.25	−0.50 *
Mg	0.73 *	0.69 *	−0.92 *	0.60 *	1.00 *	0.31 *	0.64 *	0.90 *	0.43 *	−0.47 *	0.57 *	0.54 *	−0.46 *	−0.46 *	−0.60 *
Mn	0.35 *	0.26	−0.31 *	0.37 *	0.31 *	1.00 *	0.26	0.48 *	0.10	−0.09	0.56 *	0.15	−0.18	−0.21	−0.24
Cu	0.58 *	0.49 *	−0.64 *	0.53 *	0.64 *	0.26	1.00 *	0.70 *	0.29	−0.56 *	0.53 *	0.54 *	−0.30	−0.24	−0.44 *
Zn	0.83 *	0.74 *	−0.95 *	0.71 *	0.90 *	0.48 *	0.70 *	1.00 *	0.38 *	−0.52 *	0.73 *	0.61 *	−0.44 *	−0.43 *	−0.70 *
Be	0.14	0.33 *	−0.39 *	0.27	0.43 *	0.10	0.29	0.38 *	1.00 *	−0.25	0.27	0.10	−0.19	−0.21	−0.08
As	−0.32 *	−0.24	0.54 *	−0.48 *	−0.47 *	−0.09	−0.56 *	−0.52 *	−0.25	1.00 *	−0.26	−0.21	0.27	0.19	0.47 *
Cr	0.67 *	0.56 *	−0.58 *	0.36 *	0.57 *	0.56 *	0.53 *	0.73 *	0.27	−0.26	1.00 *	0.38 *	−0.11	−0.37 *	−0.40 *
Mo	0.68 *	0.45 *	−0.57 *	0.53 *	0.54 *	0.15	0.54 *	0.61 *	0.10	−0.21	0.38 *	1.00 *	−0.24	−0.13	−0.51 *
Ti	−0.31 *	−0.32 *	0.45 *	−0.40 *	−0.46 *	−0.18	−0.30	−0.44 *	−0.19	0.27	−0.11	−0.24	1.00 *	0.28	0.20
Tl	−0.31 *	−0.38 *	0.42 *	−0.25	−0.46 *	−0.21	−0.24	−0.43 *	−0.21	0.19	−0.37 *	−0.13	0.28	1.00 *	0.20
Pb	−0.69 *	−0.42 *	0.67 *	−0.50 *	−0.60 *	−0.24	−0.44 *	−0.70 *	−0.08	0.47 *	−0.40 *	−0.51 *	0.20	0.20	1.00 *

Na, sodium; K, potassium; Ca, calcium; P, phosphorus; Mg, magnesium; Mn, manganese; Cu, copper; Zn, zinc; Be, beryllium; As, arsenic; Cr, chromium; Mo, molybdenum; Ti, titanium; Tl, thallium; Pb, lead; *, *p* < 0.05.

**Table 19:** Univariate Regression Analyses of Clinical Factors Associated with Element Concentrations in Ovarian Cancer Tissue.

Element	Treatment (B vs. A) β	Age β	BMI β	Menopause β	Diabetes β	Smoking β
Na	−0.62 ***	−0.21 *	−0.74 ***	−0.09	−0.18	−0.11
K	−0.55 ***	−0.08	−0.42 **	−0.07	−0.09	+0.05
Ca	+0.68 ***	+0.27 **	+0.94 ***	+0.11	+0.15	+0.04
P	−0.48 ***	−0.18	−0.21	+0.02	−0.20	−0.07
Mg	−0.61 ***	+0.06	−0.82 ***	−0.13	−0.19	−0.10
Mn	−0.66 ***	−0.12	−0.35 *	−0.06	−0.08	−0.05
Cu	−0.70 ***	−0.16	−0.39 *	−0.17	−0.18	−0.06
Zn	−0.58 ***	−0.85 ***	−0.33	−0.12	−0.03	−0.07
Be	−0.12	+0.05	−0.15	−0.14	−0.02	−0.04
As	+0.36 **	+0.08	+0.24	+0.12	+0.20	+0.09
Cr	−0.69 ***	−0.11	−0.28	+0.08	−0.19	−0.08
Mo	−0.47 ***	−0.29 *	−0.22	−0.14	−0.11	−0.06
Ti	+0.51 ***	+0.38 **	+0.15	+0.17	+0.18	+0.05
Tl	+0.39 ***	+0.01	+0.09	+0.07	+0.04	+0.03
Pb	+0.49 ***	+0.15	+0.12	−0.05	−0.01	+0.10

Na, sodium; K, potassium; Ca, calcium; P, phosphorus; Mg, magnesium; Mn, manganese; Cu, copper; Zn, zinc; Be, beryllium; As, arsenic; Cr, chromium; Mo, molybdenum; Ti, titanium; Tl, thallium; Pb, lead; β, standardized regression coefficient; *, *p* < 0.05; **, *p* < 0.01; ***, *p* < 0.0001.

**Table 20:** Multivariate Regression Analyses Adjusted for Group, Age, BMI, Menopause, Smoking, and Diabetes.

Element	Group B (95% CI)	Age B (95% CI)	BMI B (95% CI)	Menopause B (95% CI)	Diabetes B (95% CI)	Smoking B (95% CI)	R^2^
Na	−47.55 (−64.41 to −30.68)	−0.35 (−0.86 to 0.16)	−0.73 (−1.41 to −0.05)	5.41 (−9.33 to 20.14)	−12.72 (−39.12 to 13.69)	6.46 (−12.15 to 25.06)	0.646
K	−11.28 (−15.89 to −6.68)	0.02 (−0.12 to 0.16)	0.27 (0.08 to 0.46)	1.42 (−2.65 to 5.50)	−4.11 (−11.31 to 3.10)	3.19 (−1.89 to 8.27)	0.577
Ca	5.76 (4.83 to 6.68)	0.03 (−0.00 to 0.06)	−0.03 (−0.07 to 0.01)	−0.77 (−1.64 to 0.10)	6.26 (4.81 to 7.70)	0.27 (−0.75 to 1.29)	0.919
P	−4.01 (−5.74 to −2.28)	−0.04 (−0.09 to 0.01)	0.04 (−0.03 to 0.11)	1.41 (−1.75 to 4.57)	−2.33 (−5.04 to 0.38)	−0.23 (−2.13 to 1.68)	0.603
Mg	−1.70 (−2.13 to −1.27)	0.02 (−0.01 to 0.04)	−0.01 (−0.02 to 0.01)	−0.02 (−0.22 to 0.19)	−1.56 (−2.23 to −0.89)	0.08 (−0.39 to 0.56)	0.837
Mn	−2.76 (−3.52 to −1.99)	0.01 (−0.01 to 0.04)	0.01 (−0.02 to 0.04)	1.13 (0.03 to 2.23)	3.47 (2.27 to 4.67)	−0.06 (−0.91 to 0.79)	0.702
Cu	−0.18 (−0.24 to −0.12)	−0.00 (−0.00 to 0.00)	−0.00 (−0.01 to 0.00)	−0.05 (−0.13 to 0.02)	−0.03 (−0.12 to 0.06)	0.00 (−0.06 to 0.07)	0.624
Zn	−0.12 (−0.15 to −0.10)	−0.00 (−0.00 to 0.00)	−0.00 (−0.01 to 0.00)	−0.03 (−0.06 to 0.01)	−0.04 (−0.07 to −0.01)	−0.00 (−0.03 to 0.02)	0.873
Be	−0.27 (−0.63 to 0.09)	−0.01 (−0.03 to 0.01)	−0.01 (−0.03 to 0.01)	−0.10 (−0.41 to 0.21)	−0.17 (−0.74 to 0.40)	0.31 (−0.09 to 0.71)	0.225
As	0.000 (0.000 to 0.000)	0.000 (0.000 to 0.000)	0.000 (0.000 to 0.000)	0.000 (−0.000 to 0.000)	0.000 (−0.000 to 0.000)	0.000 (−0.000 to 0.000)	0.414
Cr	−2.83 (−3.57 to −2.08)	0.02 (−0.01 to 0.05)	0.01 (−0.02 to 0.04)	1.31 (0.32 to 2.29)	1.53 (0.37 to 2.69)	0.26 (−0.55 to 1.08)	0.670
Mo	−0.08 (−0.12 to −0.04)	−0.00 (−0.00 to 0.00)	−0.00 (−0.01 to 0.00)	−0.02 (−0.08 to 0.04)	−0.03 (−0.10 to 0.03)	−0.03 (−0.08 to 0.01)	0.411
Ti	0.58 (0.01 to 1.15)	0.01 (−0.01 to 0.03)	0.02 (−0.01 to 0.05)	0.28 (−0.34 to 0.89)	0.61 (−0.28 to 1.50)	0.30 (−0.33 to 0.92)	0.340
Tl	0.05 (0.01 to 0.08)	−0.00 (−0.00 to 0.00)	0.00 (−0.00 to 0.00)	0.01 (−0.03 to 0.05)	0.02 (−0.03 to 0.08)	−0.01 (−0.04 to 0.03)	0.330
Pb	0.17 (0.10 to 0.24)	−0.00 (−0.01 to 0.01)	−0.00 (−0.01 to 0.01)	−0.10 (−0.24 to 0.04)	0.16 (0.04 to 0.27)	−0.02 (−0.10 to 0.06)	0.558

Na, sodium; K, potassium; Ca, calcium; P, phosphorus; Mg, magnesium; Mn, manganese; Cu, copper; Zn, zinc; Be, beryllium; As, arsenic; Cr, chromium; Mo, molybdenum; Ti, titanium; Tl, thallium; Pb, lead; B, unstandardized regression coefficient; 95%Cl, 95% confidence interval; R^2^, coefficient of determination.

## Data Availability

The data used to support the findings of this study are included in the article.

## Supplementary Materials

The following supporting information can be downloaded at: https://www.mdpi.com/article/10.3390/cancers18071051/s1, Supplementary Figure S1: Distribution of macronutrient element concentrations (Na, K, Ca, P, Mg) across endometrial cancer subgroups (C, G1, G2, G3); Supplementary Figure S2: Distribution of trace and toxic element concentrations (Mn, Cu, Zn, Cr, Mo, Ti, Tl, Pb, Be, As) across endometrial cancer subgroups (C, G1, G2, G3); Supplementary Figure S3: Distribution of macronutrient element concentrations (Na, K, Ca, P, Mg) across ovarian cancer groups (A, B, C); Supplementary Figure S4: Distribution of trace and toxic element concentrations (Mn, Cu, Zn, Cr, Mo, Ti, Tl, Pb, Be, As) across ovarian cancer groups (A, B, C).

## Abbreviations

The following abbreviations are used in this manuscript:

ANOVA	Analysis of Variance
As	Arsenic
Be	Beryllium
BMI	Body Mass Index
C	Control group
Ca	Calcium
Cd	Cadmium
Cr	Chromium
Cu	Copper
DNA	Deoxyribonucleic Acid
EC	Endometrial Cancer
Fe	Iron
G1	Well-differentiated tumor grade
G2	Moderately differentiated tumor grade
G3	Poorly differentiated tumor grade
HbA1c	Glycated Hemoglobin
HCl	Hydrochloric Acid
ICP–OES	Inductively Coupled Plasma Optical Emission Spectrometry
IARC	International Agency for Research on Cancer
K	Potassium
kg/m^2^	Kilograms per Square Meter
Mg	Magnesium
Mn	Manganese
Mo	Molybdenum
Na	Sodium
Ni	Nickel
OC	Ovarian Cancer
P	Phosphorus
Pb	Lead
ROS	Reactive Oxygen Species
Se	Selenium
TCGA	The Cancer Genome Atlas
Ti	Titanium
Tl	Thallium
Zn	Zinc
