# Supplementary material for: Assessment of Essential and Toxic Element Levels in Endometrial and Ovarian Cancer
Source: Cancers (Basel). 2026 Mar 24;18(7):1051. doi: 10.3390/cancers18071051 (PMC13072392; doi:10.3390/cancers18071051)
Supplement: Supplementary file 1 [file cancers-18-01051-s001.zip › cancers-4217333-supplementary.pdf]

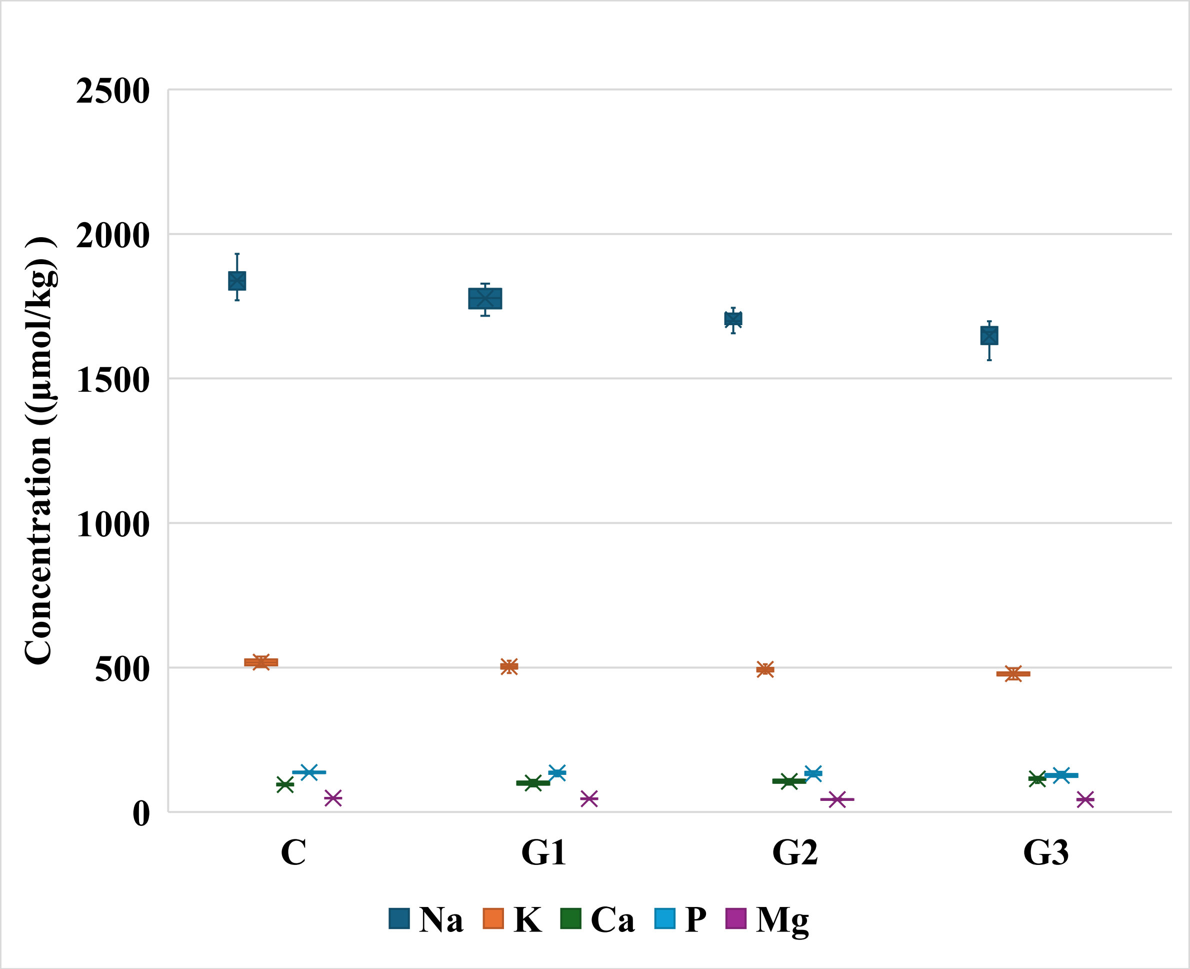

Supplementary Figure S1. Distribution of macronutrient element concentrations (Na, K, Ca, P, Mg) across endometrial cancer subgroups (C, G1, G2, G3)

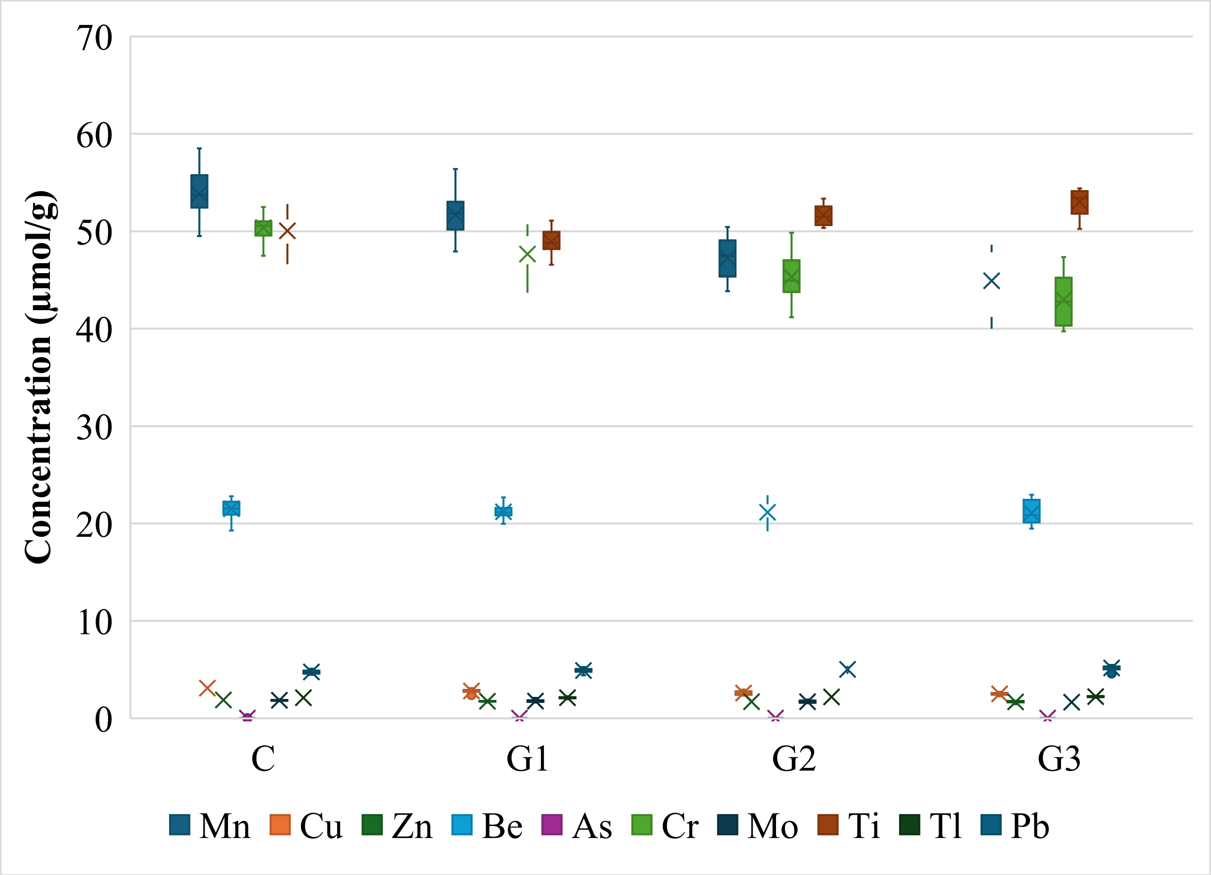

Supplementary Figure S2. Distribution of trace and toxic element concentrations (Mn, Cu, Zn, Cr, Mo, Ti, Tl, Pb, Be, As) across endometrial cancer subgroups (C, G1, G2, G3)

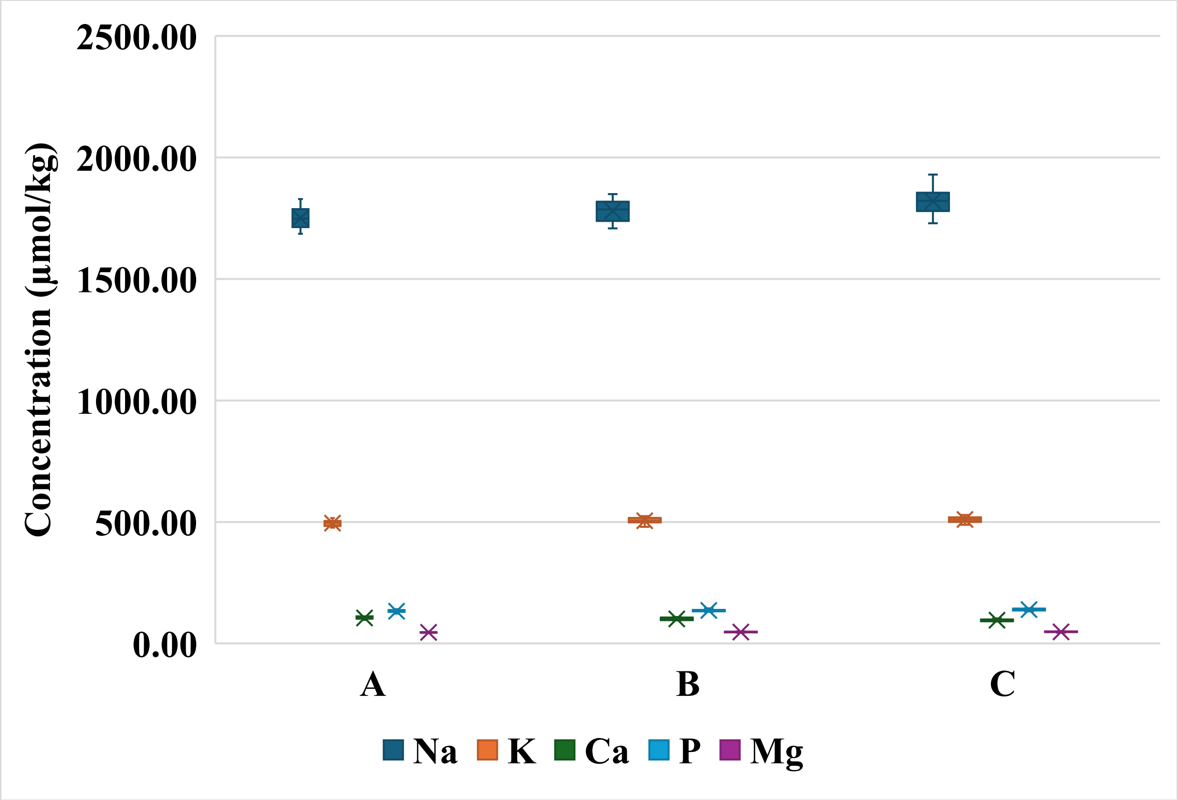

Supplementary Figure S3. Distribution of macronutrient element concentrations (Na, K, Ca, P, Mg) across ovarian cancer groups (A, B, C)

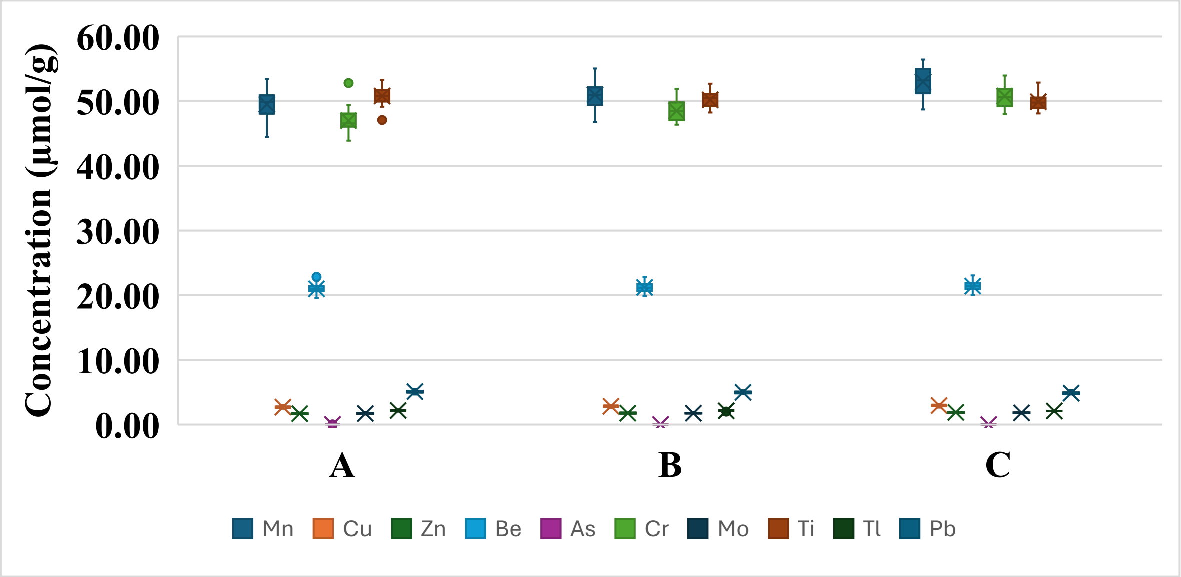

Supplementary Figure S4. Distribution of trace and toxic element concentrations (Mn, Cu, Zn, Cr, Mo, Ti, Tl, Pb, Be, As) across ovarian cancer groups (A, B, C)
